# Characterization of Glycolytic Enzymes - rAldolase and rEnolase of *Leishmania donovani,* Identified as Th1 Stimulatory Proteins, for Their Immunogenicity and Immunoprophylactic Efficacies against Experimental Visceral Leishmaniasis

**DOI:** 10.1371/journal.pone.0086073

**Published:** 2014-01-24

**Authors:** Reema Gupta, Vikash Kumar, Pramod Kumar Kushawaha, Chandradev Pati Tripathi, Sumit Joshi, Amogh Anant Sahasrabuddhe, Kalyan Mitra, Shyam Sundar, Mohammad Imran Siddiqi, Anuradha Dube

**Affiliations:** 1 Divisions of Parasitology, CSIR-Central Drug Research Institute, Lucknow, India; 2 Molecular and Structural Biology, CSIR-Central Drug Research Institute, Lucknow, India; 3 Electron Microscopy, CSIR-Central Drug Research Institute, Lucknow, India; 4 Department of Medicine, Institute of Medical Sciences, Banaras Hindu University, Varanasi, India; Centro de Investigacion y de Estudios Avanzados del Instituto Politecnico Nacional, Mexico

## Abstract

Th1 immune responses play an important role in controlling Visceral Leishmaniasis (VL) hence, *Leishmania* proteins stimulating T-cell responses in host, are thought to be good vaccine targets. Search of such antigens eliciting cellular responses in Peripheral blood mononuclear cells (PBMCs) from cured/exposed/*Leishmania* patients and hamsters led to the identification of two enzymes of glycolytic pathway in the soluble lysate of a clinical isolate of *Leishmania donovani* - Enolase (LdEno) and aldolase (LdAld) as potential Th1 stimulatory proteins. The present study deals with the molecular and immunological characterizations of LdEno and LdAld. The successfully cloned and purified recombinant proteins displayed strong ability to proliferate lymphocytes of cured hamsters’ along with significant nitric-oxide production and generation of Th1-type cytokines (IFN-γ and IL-12) from stimulated PBMCs of cured/endemic VL patients. Assessment of their prophylactic potentials revealed ∼90% decrease in parasitic burden in rLdEno vaccinated hamsters against *Leishmania* challenge, strongly supported by an increase in mRNA expression levels of iNOS, IFN-γ, TNF-α and IL-12 transcripts along with extreme down-regulation of TGF-β, IL-4 and IL-10. However, animals vaccinated with rLdAld showed comparatively lesser prophylactic efficacy (∼65%) with inferior immunological response. Further, with a possible implication in vaccine design against VL, identification of potential T-cell epitopes of both the proteins was done using computational approach. Additionally, *in-silico* 3-D modelling of the proteins was done in order to explore the possibility of exploiting them as potential drug targets. The comparative molecular and immunological characterizations strongly suggest rLdEno as potential vaccine candidate against VL and supports the notion of its being effective T-cell stimulatory protein.

## Introduction

The search for an effective vaccine against Visceral Leishmaniasis (VL), caused by *Leishmania donovani* producing a severe and potentially fatal systemic disease in which parasites invade the macrophages of liver, spleen and bone marrow causing serious illness, remains a challenging and elusive goal. VL is a major public health importance in Indian subcontinent with more than 90% of the world’s cases and affects the poorest population mainly in rural areas. Emergence of resistance against pentavalent antimony, the mainstay of treatment, in north-eastern India and the toxicity, availability and affordability of second-line drugs (pentamidine and amphotericin B) leaves the situation more complicated. Human VL is characterized by a marked humoral response and impaired cell-mediated immunity (CMI), associated with an inability to control infection. During VL infection, impairment of nitric oxide generation and IL-12 production from macrophages occurs whereas the disease promoting cytokines TGF-β and IL-10 are enhanced [1], [2]. Still, it has been demonstrated that the ability to mount a pro-inflammatory response is critical for control and eventual resolution of infection and that the Th1 cytokines IL-2, IL-12 and IFN-γ are essential for response [3], [4]. Because of the lack of effective and low-cost treatments and the irreversibility of tissue damage during infection, considerable attention has been focused towards vaccine development. Recent research on leishmaniasis has been focused towards determining strategies that specifically stimulate protective immune responses in the absence of those that may cause pathology and/or interfere with protection [5]. Thus, leishmanial antigens that predominantly stimulate Th1 responses in patient cells or rodents infected with the parasite have been accepted as ‘potential protective antigens’ and therefore promising vaccine candidates. Earlier studies in our laboratory, by using classical activity based fractionation and sub-fractionation of the soluble proteins from clinical isolate of *L. donovani* promastigote, led to the identification of a potent sub-fraction ranging from 89.9 to 97.1 kDa which induced Th1 type cellular responses in cured *Leishmania* patients and hamsters along with significant prophylactic efficacy in hamsters [6]. Further proteomic characterization of this sub-fraction led to the identification of 18 Th1 stimulatory proteins and among them Enolase and Fructose bisphosphate Aldolase (FBA), the vital proteins belonging to the glycolytic pathway, were also present. Aldolase is a central glycolytic enzyme (E.C. 4.1.2.13) in carbohydrate metabolism, catalyzing the cleavage of fructose 1,6-bisphosphate into two triose sugars, glyceraldehyde 3-phosphate and dihydroacetone phosphate [7] whereas Enolase (2-phospho-D-glycerate hydrolase, EC 4.2.1.11) is known to catalyse the reversible dehydration of D-2-phosphoglycerate (2PGA) to phosphoenolpyruvate (PEP) in both glycolysis and gluconeogenesis [8].

During the past decade glycolytic enzymes have emerged as vaccine targets/candidates in many different organisms. This has been attributed because of their moonlighting functions associated along with their classical functions in glycolysis. In *Fasciola hepatica* [9] and *Schistosoma mansoni* [7], recombinant FBA was confirmed to provide significant protection for experimentally infected animals. Further, FBA was immuno-screened with sera from patients infected with *Onchocerca volvulus* [10], *S. mansoni* [11], *Streptococcus pneumonia* [12] and *Plasmodium falciparum* [13]. It is also considered as a potential diagnostic antigen in case of human giardiasis [14]. A unique β-(Man)3-FBA conjugate was developed by Xin *et al*, wherein protection against *C. albicans* infection was uniquely acquired through immunity against the carbohydrate and the FBA peptide [15]. In *Leishmania*, apart from its glycolytic function Enolase also localizes on the surface and binds plasminogen [16], [17] which contributes to the virulence of the parasite [18]. Therefore, these are vital for energy production that is necessary for parasite activities and survival. An anti-enolase antibody was observed to interfere with plasminogen binding thus making this a candidate vaccine antigen [18]. Enolase has been reported as vaccine candidate in *Plasmodium sp* [19] and other microorganisms such as *Candida albicans* [20], [21], [22], *Chlamydia pneumonia* [23] and *Streptococcus sp* [24], [25]. There have been reports regarding the presence of anti-enolase antibodies among malaria patients [26] and prophylactic potential of recombinant *P. falciparum* enolase in mice against a challenge with a lethal strain of *P. yoelii* suggested that enolase is a potential protective antigen [19]. Since there has been no report of enolase as well as aldolase as vaccine candidates in case of *Leishmania*, it was pertinent to assess their immunogenic and prophylactic potential against VL- a fatal infection. Therefore, in the present study we undertook (1) molecular cloning and characterization of enolase as well as aldolase, (2) *in vitro* cellular responses of both proteins using lymphocytes/PBMCs of cured *Leishmania* hamsters/patients and (3) evaluation of prophylactic efficacy against *L. donovani* infection in golden hamster (*Mesocricetus auratus*), since this is the perfect experimental model for VL as it closely mimics the situation as found in human VL (4) bioinformatic approach for predicting T-cell epitopes in both the proteins.

## Materials and Methods

### Ethics Statement

All animal care and experimental use conformed to Committee for the Purpose of Control and Supervision on Experiments on Animals guidelines for laboratory animal facilities and were approved by the Committee on the Ethics of Animal Experiments of Central Drug Research Institute (Approval Number: 154/10/Para/IAEC dated 04.10.10.27-2956). The protocol and study with patients’ was approved by the Ethics committee of the Kala-azar Medical Research Centre, Muzaffarpur (Protocol # EC-KAMRC/Vaccine/VL/2007-01) and written informed consent was obtained from patients before enrolment to this study. All the human subjects underwent clinical examination by a local physician for leishmanial and other possible infections.

### Animals, Parasites and Cell Lines

Laboratory-bred male golden hamsters (*Mesocricetus auratus*, 45–50 g) from the Institute’s animal house facility were used as experimental host. They were housed in a climatically controlled room and fed with standard rodent food pellet (Lipton India, Mumbai, India) and water *ad libitum*. The *L. donovani* clinical strain procured from a patient admitted to the Kala-azar Medical Research Centre, Institute of Medical Sciences, BHU, Varanasi, was cultured *in vitro* as described elsewhere [27] and has also been maintained in Golden hamsters through serial passage, i.e. from amastigote to amastigote [28]. Mouse macrophage cell line J774A.1, procured from Tissue Culture Facility of the institute was maintained in RPMI-1640 through serial passage in 75 cm^2^ culture flasks at 37°C and 5% CO_2_. The confluent cells were harvested using cell scrapper for the estimation of nitric oxide (NO) production.

### Preparation of Soluble *Leishmania donovani* Promastigote Antigen

Soluble *L. donovani* promastigote antigen (SLD) was prepared as per method described by Gupta *et al* [29]. Briefly, 10^9^ log phase promastigotes harvested from 3 to 4 days of culture was washed four times in cold phosphate-buffered saline (PBS) and resuspended in PBS containing protease inhibitors cocktail (Sigma). It was then subjected to ultrasonication and centrifugation at 40,000×g for 30 min. The protein content of the supernatant was estimated [30] and stored at −80°C until use.

### Cloning, Expression and Purification of Recombinant Proteins

Gene specific primers were designed according to the *L. major* sequence available in the database and are as follows: Enolase, forward 5′-GGATCCATGCCGATCCGAAAGGTTTACGCC-3′ and reverse 5′-GAATTCTTACGCCCAGCCGGGGTAGCCGTA-3′; Aldolase, forward 5′-GGATCCATGTCGCGTGTGACCATCTTTCA-3′ and reverse 5′- GAATTCTTAATAAGTGTTGCCTTTCACGTAC-3′containing *BamHI* and *EcoRI* sites (underlined) respectively. Both the genes were amplified using 2 X PCR Master mix (Fermentas) and the amplified PCR product was inserted in-frame with a 6xHis tag into the *BamHI*/*EcoRI* site of the vector pET-28a (Novagen, USA) and the insert was sequenced. This clone was used to express the *L. donovani* Enolase in *Escherichia coli* C41 (DE3) strain. The recombinant protein (rLdEno), containing an N-terminal His-tag, was purified using Ni-NTA agarose column (Qiagen), following standard procedures and lysis buffer as 50 mM Tris-Cl, 2 mM MgSO_4_, 1 mM KH_2_PO_4_, 75 mM KCl, 10 mM Imidazole and 10% glycerol, pH 8.0.

Similarly recombinant aldolase (rLdAld) was purified using lysis buffer as 50 mM Tris buffer, 300 mM NaCl, 10 mM imidazole and 10% glycerol, pH 8.0.

The eluted proteins were analyzed by 12% SDS–PAGE, stained with coomassie brilliant blue stain (Sigma) and the protein content of the fractions was estimated by the Bradford method using bovine serum albumin (BSA) as standard. The lipopolysaccharides (LPS) content of the recombinant proteins was measured by *Limulus* amoebocyte lysate test (QCL-1000, Lonza).

### Production of Polyclonal Antibodies against Recombinant Proteins for Western Blot Analysis

Rabbits were immunized with 50 µg of purified proteins (rLdEno and rLdAld) with Freund’s complete adjuvant for the first immunization and then boosted three times with recombinant protein in Freund’s incomplete adjuvant. Serum was obtained 7 days after the last booster and antibody titres were determined by ELISA.

For immunoblots, equal amount of whole cell lysate and SLD of *L. donovani* in each lane was subjected to SDS-PAGE and electrophoretically transferred to a nitrocellulose membrane using a semi-dry blot apparatus Hoefer Semiphor (Pharmacia Biotech). After overnight blocking in 5% BSA, the membrane was incubated with antiserum to recombinant protein at a dilution of 1∶10,000 for 120 min at room temperature (RT). The membrane was then probed with HRP-conjugated goat anti-rabbit IgG (Banglore Genei). Finally, the blot was developed by using diaminobenzidine, imidazole and H_2_O_2._

### Enzyme Kinetics

Enolase activity was measured directly by monitoring the formation of Phosphoenol pyruvate (PEP) using SPECTRAmax PLUS 384 (Molecular Devices Corp.) at 240 nm as described earlier [31]. The assay was performed at 25°C in a 1.0 mL reaction mixture containing 20 mM Tris–HCl, pH 7.5, 2 mM MgSO4, 75 mM KCl and 1 mM 2-phosphoglycerate (2PGA) or 2 mM PEP. For the determination of the values of kinetic parameters, the substrate concentrations were varied from 0 to 10 mM for 2PGA and 0 to 7.5 mM for PEP.

Aldolase activity was measured by using hydrazine assay modified by Jagannathan *et al* [32] using D-Fru-1,6-P2 (Sigma) as substrate and hydrazine sulfate (Sigma) as detection reagent for the formed 3-phosphoglyceraldehyde. The assay was performed in a 1.0 mL reaction mixture and the absorbance was recorded at 240 nm. To calculate Vmax and Km, the activity was measured between 0.1 and 7 mM D-Fru-1, 6-P2.

### Immunolocalization

*L. donovani* promastigotes were washed in PBS, fixed with paraformaldehyde and allowed to adhere to poly-(L-lysine)-coated coverslips. Cells were then permeabilized with 0.2% (v/v) Triton X-100 and washed again with PBS. The fixed cells were incubated in PBS containing 3% BSA for 30 min and washed again. Then, the cells were incubated with primary antibodies (rabbit anti-*L. donovani* enolase/anti- *L. donovani* aldolase) at a dilution 1∶2000 for 1 h, rinsed with PBS and incubated again for 1 h with FITC conjugated goat anti-rabbit IgG. The coverslips were mounted on clean slides using 10 µl of Flourescent mounting media (Merck) and finally observed under confocal laser scanning microscope (Zeiss LSM 510 Meta) using 60×oil immersion plan apochromate objective lens (NA 1.4). The negative control samples were processed in parallel by omitting primary antibodies that were used to negate background fluorescence, if any, by adjusting laser power and gain/offset settings before image acquisition. Images were processed in Adobe Photoshop (version 7.1) for presentation purposes.

### *In-silico* Protein Modeling and Analysis

Keeping in mind that these proteins (Enolase and aldolase) may be potential drug targets, we attempted 3D-structural investigation on homology modeled *L. donavani* enolase and aldolase. Search for suitable templates for modeling was carried out using blastP tool [www.blast.ncbi.nlm.nih.gov/Blast.cgi?PAGE=Proteins] against Protein data Bank [www.rcsb.org/pdb]. For LdAld modeling we selected crystal structure of *L. mexicana* aldolase (1EPX) and human gamma aldolase (2ALD) as templates [33], [34]. LdEno model was based on crystal structure of *Trypansoma brucei* enolase (1OEP) [35]. Modeller9v10 [36], [37] was used to generate ten models of each protein and best model was chosen on the basis of Ramachandaran plot and stereochemical properties calculated by SAVS server [www.nihserver.mbi.ucla.edu/SAVES/]. UCSF chimera [38] was used for model visualization and other structural analysis.

### T-cell Immune Response

#### Patients and isolation of Peripheral Blood Mononuclear Cells (PBMCs)

The study was based on a convenience human sample of 22 belonging to the following categories/groups:

1. Seven treated cured patients (4 males and 3 females, age ranging from 5–40 years) from hyper-endemic areas of Bihar. All the patients had received complete course of amphotericin B and had recovered from VL. Samples were collected from 2 months to 1 year after the completion of treatment. Diagnosis was established in all cases by demonstration of parasites in splenic aspirates and found negative at the time of study.
2. Five endemic household contacts (2 males and 3 females, age range-15 to 45 years) belonging to the family of infected or cured patients, that neither showed clinical symptoms nor received any treatment for Kala-azar.
3. Five infected patients (3 males and 2 females, age range−5 to 40 years) showing clinical symptoms of Kala-azar which was confirmed by the presence of parasite in the splenic aspirate.
4. Five normal healthy donors (3 males and 2 females, age range 25–30 years) from nonendemic areas, without any history of leishmaniasis, served as negative control. Heparinized venous blood (10 ml each) was collected from all the study subjects and mixed with equal amount of PBS (1.5 M, pH 7.4). The suspension was then layered onto Ficoll-Hypaque (Histopaque 1077, Sigma) in a 2∶1 ratio and subjected to density gradient centrifugation. The isolated PBMCs were washed three times with incomplete RPMI medium and a final suspension of 1×10^6^ cells/ml was made in complete RPMI medium (cRPMI) after determining cell viability by trypan blue staining method. These were used for various immunological assays.

#### Assessment of Lymphocyte proliferative responses in cured/exposed patients and hamsters

Hamsters infected intracardially with 10^7^ amastigotes were assessed one month later for parasitic burden by splenic biopsy as described earlier [39]. The splenic dab-smears were fixed in methanol, stained with Giemsa, and the number of amastigotes/1000 cell nuclei was counted. The animals harboring 25–30 amastigotes/100 macrophage cell nuclei were then treated orally with antileishmanial drug-Miltefosine (Zentaris, Germany) at 40 mg/kg body weight daily for 5 consecutive days. The treated animals were then reassessed for complete cure by biopsy after 30 day post-treatment. Mononuclear cells were separated from lymph nodes (inguinal and mesenteric) of cured (Day 30 p.t.), infected (Day 30 p.i.) as well as normal hamsters following the protocol of Garg *et al.* [27] and a suspension of 10^6^ cells/ml was made in cRPMI. These cells were employed for lymphoproliferative assay and for the estimation of NO production.

PBMCs suspension (1×10^6^ cells/ml) of cured/exposed patients and lymphocytes of normal, infected and cured hamsters was cultured in 96-well flat bottom tissue culture plates. This assay was carried out as per protocol described earlier [39]. About 100 µl of predetermined concentration (10 µg/ml) of Phytohaemagglutinin (PHA, Sigma) for Patient’s PBMCs, Concavalin A (Con A) for hamster’s lymphocytes, as well as rLdEno, rLdAld and SLD were added to the wells in triplicate. Wells without stimulants served as blank controls. Cultures were incubated at 37°C and 5% CO_2_ in a CO_2_ incubator for 3 days in case of mitogens, and for 5 days in case of antigens (rLdAld/rLdEno/SLD). Eighteen hours prior to termination of experiment, 50 µl of XTT (Roche diagnostics) was added to 100 µl of supernatants of each well and optical density at 480 nm was determined by using 650 nm as the reference wavelength.

#### Estimation of NO activity in macrophages of hamsters and cell lines

Isolated lymphocytes from all the three study groups of hamsters’ viz. normal, infected and cured, were suspended in cRPMI and plated at 10^5^ cells/well and stimulated for 3 days in case of LPS and 5 days in case of rLdEno/rLdAld and SLD at 10 µg/ml. The supernatants of stimulated lymphocytes were co-cultured with the peritoneal macrophages of naive hamsters [27] as well as macrophage cell line J774 A.1. The presence of NO was assessed in the culture supernatants (100 µl) collected from the exposed macrophage cultures (24 h after incubation) by adding equal volume of Griess reagent (Sigma). The content was left for 10 min at RT and the absorbance of the reaction was measured at 540 nm in an ELISA reader [40]. The nitrite concentration in the macrophages culture supernatant samples was extrapolated from the standard curve plotted with sodium nitrite. The same protocol for measuring NO production was used for vaccination study.

#### Assessment of cytokine levels- IFN-γ/IL-12/IL-10 in lymphocytes of cured/endemic patients

With the aim of investigating the Th1/Th2 stimulatory potential of rLdEno and rLdAld we further assessed the cytokine levels viz. IFN-γ and IL-12p40 as well as IL-10 in PBMCs from cured patients as well as in endemic contacts. Culture of PBMCs (1×10^6^ cells/ml) was set up in 96-well culture plates and PHA, SLD and rLdEno/rLdAld were added at a concentration of 10 µg/ml in triplicate wells. The levels of IFN-γ, IL-12 as well as IL-10 were estimated by using commercial ELISA kits according to the manufacturer’s protocol (BD Biosciences Pharmingen). The results were expressed as picograms of cytokine/ml, based on the standard curves generated using a recombinant cytokine provided in the kit. The lower detection limits for various cytokines were as follows: 4.7 pg/ml for IFN-γ, 7.8 pg/ml for IL-12p40 and 7.0 pg/ml for IL-10.

### Protection Studies following Vaccination of Hamsters with rLdEno as Well as rLdAld

Experiments were performed on five groups of hamsters (12–15 per group), wherein groups 1–3 served as controls as described below and group 4 and 5 as the main experimental groups: group 1, unvaccinated and unchallenged (normal control); group 2, unvaccinated and challenged (infected control); group 3, BCG alone; group 4, vaccinated with rLdEno+BCG (vaccinated group); and group 5, vaccinated with rLdAld+BCG. The hamsters of Group 4 and 5 were immunized i.d on the back with rLdEno/rLdAld (50 µg/50 µl per animal) along with equal volume of BCG (0.1 mg/50 µl per animal) in emulsified form and group 3 was given BCG only. Fifteen days later a booster dose of half of the amount of rLdEno/rLdAld along with BCG was given i.d to all the hamsters Group 4 and 5 while only BCG to group 3.

Twenty-one days after the booster dose, all the vaccinated and unvaccinated control groups except the normal control group were challenged intracardially with 10^8^ late log phase promastigotes of *L. donovani*. The prophylactic efficacies of rLdEno as well as rLdAld were assessed in spleen, liver and bone marrow of three vaccinated hamsters on necropsy at different time intervals, i.e. on days 0, 45, 60, 90, 120 post-challenge (p.c.). Peritoneal exudates cells, inguinal lymph nodes and blood were also collected at these time points to obtain cells and sera for evaluation of cellular and antibody responses [41]. The parasitic load was assessed as the number of amastigotes/1000 cell nuclei in each organ in comparison to the unvaccinated controls. The prophylactic efficacy was calculated as the percentage inhibition (PI) of parasite multiplication using the following formula [42]:

PI = (No. of parasite from infected control−No. of parasite from vaccinated group)×100.

No. of parasite count from infected control.

#### Measurement of body, spleen, and liver weight of vaccinated hamsters

The body weight and that of the spleen and liver (on necropsy) of hamsters of all of the experimental groups were assessed at different time intervals, i.e., on days 0, 45, 60, 90, 120, and 180 p.c.

#### Measurement of delayed type hypersensitivity in hamsters

Delayed Type Hypersensitivity (DTH) was performed by injecting 50 µg/50 µl of SLD in PBS i.d into one footpad and PBS alone into the other one of each of the vaccinated and unvaccinated controls. The response was evaluated 48 h later by measuring the difference in footpad swelling between the two footpads with and without SLD for each animal [6].

#### Estimation of expression of mRNA cytokines by real-time PCR in vaccinated hamsters

To investigate the type of immune response generated upon vaccination by rLdEno as well as rLdAld, qRTPCR was performed to assess the expression of mRNAs for various cytokines and iNOS in splenic cells. For this, splenic tissues from each of the three individual animals randomly chosen from different groups at different time intervals were taken and total RNA was isolated using TRI-Reagent (Sigma). Upon quantification by using Gene-quant (Bio-Rad), one microgram of total RNA was used for the synthesis of cDNA using a first-strand cDNA synthesis kit (Fermentas). The primers for qRT-PCR, were designed using Beacon Designer software (Bio-Rad) on the basis of cytokines and iNOS mRNA sequences available on PubMed [43] (Table-1). Real-time PCR was performed as described earlier [41] using 12.5 µl of SYBR green PCR master mix (Bio-Rad), 1 µg of cDNA, and primers at a final concentration of 300 nM in a final volume of 25 µl. PCR cycles consisted of initial denaturation at 95°C for 2 min followed by 40 cycles each, of denaturation at 95°C for 30 s, annealing at 55°C for 40 s, and extension at 72°C for 40 s per cycle after which melt curve analysis was performed, using the iQ5 multicolor real-time PCR system (Bio-Rad). cDNAs from infected hamsters were used as “comparator samples” for quantification of those corresponding to test samples. The housekeeping gene Hypoxanthine phosphoribosyl transferase (HPRT) was used for normalization in all quantifications. A no-template control cDNA was also included to eliminate contaminations or nonspecific reactions. Differences in gene expressions were calculated by the comparative CT method (commonly known as delta delta CT method) [41] which compares test samples to a comparator sample and uses results obtained with a uniformly expressed control gene HPRT. The purpose of using the internal control gene i.e HPRT is to normalize the PCRs for the amount of RNA present in the two samples being compared to generate a ΔCT value. Results are expressed as the degrees of difference between ΔCT values of test and comparator samples.

**Table 1:** Sequences of forward and reverse primers of hamster cytokines used for quantitative real time RT-PCR.

S.N.	Primer	Primer sequence
1.	HGPRT	Forward 5′ GATAGATCCACTCCCATAACTG 3′
		Reverse 5′ TACCTTCAACAATCAAGACATTC 3′
2.	TNF-α	Forward 5′ TTCTCCTTCCTGCTTGTG 3′
		Reverse 5′ CTGAGTGTGAGTGTCTGG 3′
3.	IFN-γ	Forward 5′ GCTTAGATGTCGTGAATGG 3′
		Reverse 5′ GCTGCTGTTGAAGAAGTTAG 3′
4.	IL-12	Forward 5′ TATGTTGTAGAGGTGGACTG 3′
		Reverse 5′ TTGTGGCAGGTGTATTGG 3′
5.	TGF-β	Forward 5′ ACGGAGAAGAACTGCTGTG 3′
		Reverse 5′ GGTTGTGTTGGTTGTAGAGG 3′
6.	IL-4	Forward 5′ GCCATCCTGCTCTGCCTTC 3′
		Reverse 5′ TCCGTGGAGTTCTTCCTTGC 3′
7.	IL-10	Forward 5′ TGCCAAACCTTATCAGAAATG 3′
		Reverse 5′ AGTTATCCTTCACCTGTTCC 3′
8.	iNOS	Forward 5′ CGACGGCACCATCAGAGG 3′
		Reverse 5′AGGATCAGAGGCAGCACATC 3′

#### Determination of antibody levels

The levels of IgG and its isotypes – IgG1 and IgG2 in sera samples from hamsters of different groups were measured as described earlier [44]. The 96-well ELISA plates were coated with rLdEno/rLdAld (2 µg/ml in PBS) overnight at 4°C and blocked with 1.5% BSA at RT for 1 h. The sera were used at a dilution of 1∶50 for 2 h at RT. Biotin-conjugated mouse anti-Armenian and anti-Syrian hamster IgG, mouse anti-Armenian hamster IgG1 and mouse anti-Syrian hamster IgG2 (BD Biosciences Pharmingen) were added for 1 h and the plates were further incubated for 1 h with HRP-conjugated streptavidin and finally developed using OPD as substrate.

#### Post challenge survival of vaccinated hamsters

Survival of hamsters belonging to groups 4 and 5 were checked until day 180 p.c. in comparison to the normal hamsters (group 1). Animals of all the groups were given proper care and were observed for their physical conditions until their survival period. Survivals of individual hamster in each group were recorded and mean survival period was calculated.

### Mapping of T-cell Epitopes using Bioinformatics

We have used Immune epitope database (IEDB) server (www.iedb.org) for prediction of potential promiscus T cell epitopes on LdEno and LdAld. The IEDB uses information related to all experimentally determined immune epitopes [45]. It uses ANN, SMM and NetMHCpan approach to predict the potential epitopes on user submitted protein sequence. It covers wide range of MHC alleles. Here we have predicted 9-mer peptides for MHC-1 alleles and 15 mer peptides for MHC-II alleles occurring most frequently in North India. We selected promiscous epitopes having percentile score ≤45 for MHC alleles under study. BlastP search was performed to identify the similarity of predicted epitopes with human protein if any.

### Statistical Analysis

Results were expressed as mean ± S.D. The results (pooled data of three independent experiments) were analyzed by one-way ANOVA test and comparisons with control data were made with Dunnett’s post-test using Graph Pad Prism software program. Only two sets of experiments were performed for vaccination studies and in each experiment 12–15 animals were used. One-way ANOVA statistical test was used to assess the significance of the differences among various groups and a P value of <0.05 was considered significant.

## Results

### Molecular and Biochemical Characterization of LdEno and LdAld

#### Cloning, expression and purification of Enolase and aldolase

The rLdEno and rLdAld genes of *L. donovani* were successfully cloned, expressed and purified to homogeneity (Fig. S1 and S2). The gene sequences were submitted to National centre for Biotechnology Information (NCBI) with Accession Numbers EU732850 and GQ220750 (http://www.ncbi.nlm.nih.gov/nuccore/EU723850.1/GQ220750). The LPS content of the purified recombinant proteins was measured by *Limulus* amoebocyte lysate test and was shown to be below 10 endotoxin units (EU)/mg of the recombinant protein. Antibody against the recombinant proteins were raised and western blot analysis with polyclonal antiserum to rLdEno/rLdAld recognized a single specific band in the Leishmanial whole cell lysate as well as in SLD (Fig. S1 E and S2 E). However, the pre-immune sera did not react with the protein (Data not shown).

#### Enzymatic assay

Kinetic parameters were determined by varying the substrate concentrations and monitoring the changes in absorbance/minute spectrophotometrically at 240 nm. Using Michaelis Menten equation, the Kms for PGA and PEP for the enzyme Enolase were 1.54 and 3.37 mM respectively. Similarly, the Km for FBP was calculated to be 2.06 mM for the enzyme Aldolase.

#### Structural analysis of in-silico modelled LdEno and LdAld

**Figure 1:**
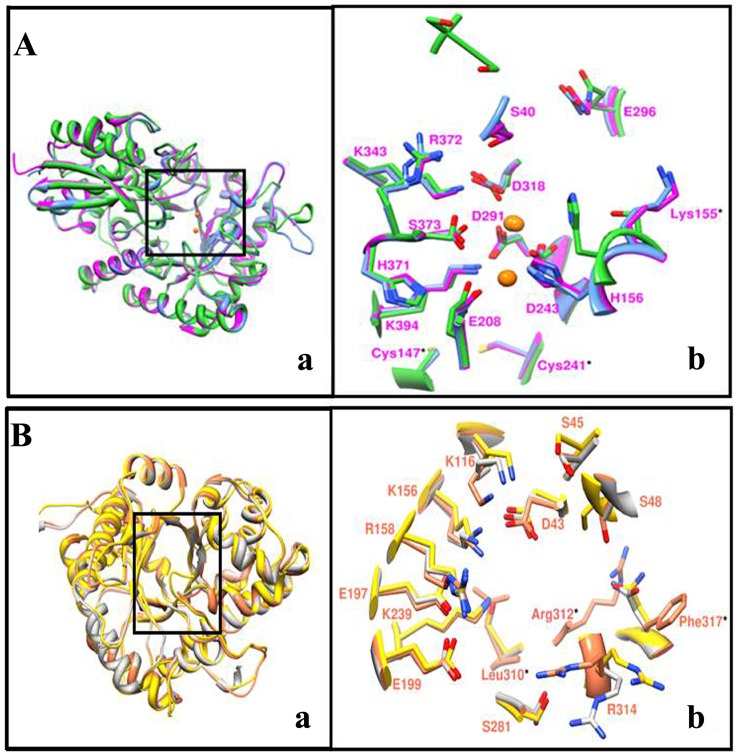
Structural overlay of *in-silico* modelled (A) LdEno (magenta), 1OEP (sky blue) and 2XSX (green) in ribbon representation (left) and (B) LdAld (coral), 1EPX (grey) and 2ALD (green) in ribbon representation (yellow). Close view of superimposed active site (right) residues in mixed representation (ribbon and stick). Fully conserved residues are labelled as single letter code while unique residues belonging to LdEno and LdAld are labelled as three letter code with star marks.

In global sequence alignment we observed significant sequence identity of LdEno and LdAld with human counterpart (Fig. S3 and S4). Different forms of Human enolase (HsEnoalpha, HsEnobeta and HsEnogamma) were compared with LdEno protein sequence and it depicted higher level of sequence identity (Fig. S3). 3D structural studies on LdEno was conducted on *in-silico* model showing 92.3% residues in core region, 7.4% residues in favoured region, 0% residues in generously allowed region and 0.3% residues in disallowed region. Structural comparison of LdEno with template (1OEP) and human muscle enolase (2XSX) revealed similar structural fold (Fig. 1 Aa). Comparison of active site residues (Ser40, His156, Asp209, Asp243, and Glu291and Asp318 in LdEno) with all three forms of human muscle enolase (alpha, beta and gamma) showed that these residues were fully conserved (Fig. S3). Near substrate binding pocket we found three differences at Cys147, Cys241 and Lys155 in LdEno (Fig. 1 Ab) with respect to human enolase having Ala, Gly and Ser respectively at same position.

Similarly, Human aldolase exists as different forms (AldA, AldB and AldC) and they have over 60% identity with each other. Sequence comparison of LdAld with each of these human Ald forms revealed conserved active site residues and its class (Fig. S4). It is reported that aldolase enzymes are classified in two classes, class I and II and they differ in their catalytic mechanism [46], [47]. We found that LdAld belongs to class I aldolase having conserved active site Lysine implicated in schiff base formation [48]. For structural analysis we selected *in-silico* generated model of LdAld showing 93.8% residues in core region, 6.2% residues in favoured region, 0% residues in generously allowed region and 0% residues in disallowed region. Comparison with human muscle aldolase structure (2ALD) showed that the LdAld shares identical (βα)_8_ barrel structural topology. Close look into active site (Fig. 1 Ba) showed that critical residues involved in substrate binding are structurally conserved with slight variation in position of side chain. However, we observed some interesting differences in the active site of LdAld with respect to HsAldA. Residues like Leu310, Val311 and Phe317 were found to be unique to LdAld (Fig. 1 Bb). These residues were substituted by Ser, Tyr, and Asn respectively in all forms of HsAld as well as other Ald sequences under study. In both sequence and structural alignment Arg312 did not share any corresponding residue, indicating that it is a unique insertion in LdAld. C-terminal region (354–374) of LdAld consisted of a loop and may have a role in the substrate binding as reported previously [49].

#### Immunolocalization

To study the subcellular location of these proteins, immunolocalization study was carried out and Enolase enzyme was localised in whole of the cytoplasm of the cell (Fig. S5 A) whereas the Aldolase enzyme was present in specialised compartments known as glycosomes- a characteristics of this parasite (Fig. S5 B). Also the Aldolase was found to be present in flagellum of the *Leishmania* promastigotes.

### Lymphoproliferative and NO Responses of Cured Hamsters in Response to rLdEno and rLdAld

**Figure 2:**
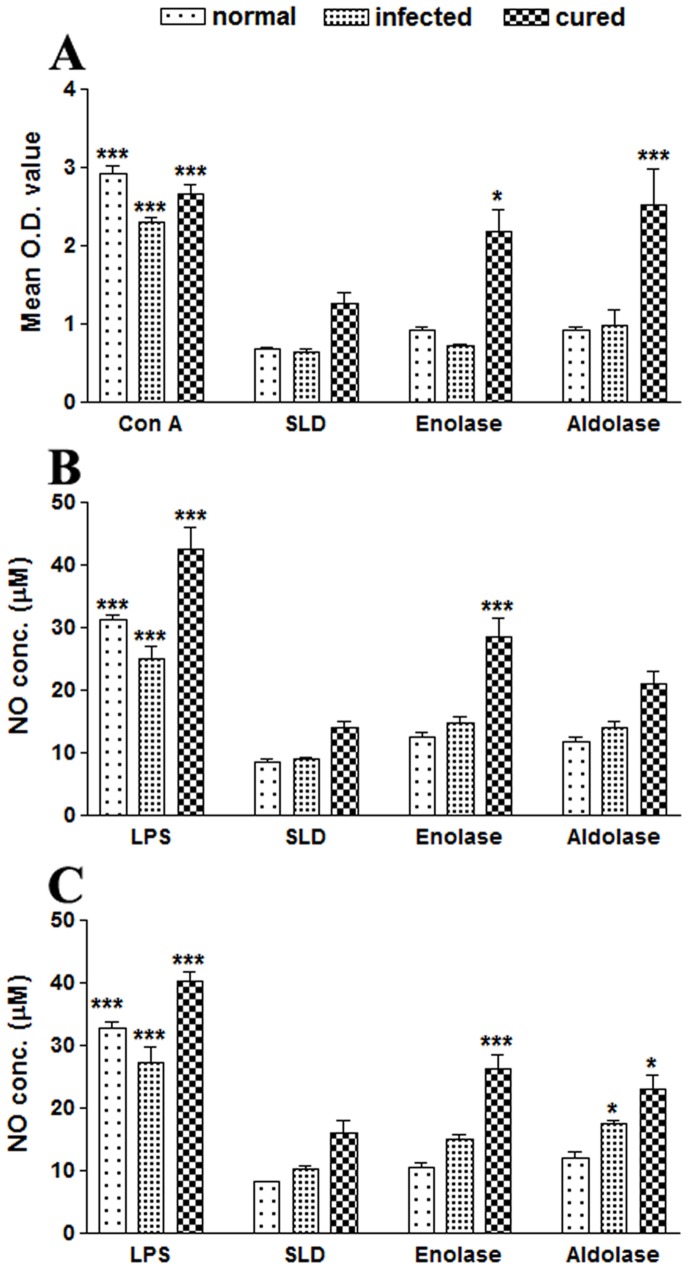
*In vitro* cellular responses in lymphocytes of cured hamsters’; LTT response in (A) lymphocytes from normal, *L. donovani* infected (30 day p.c.) and cured hamsters (30 day p.t. with miltefosine) in response to Con A, SLD, rLdEno and rLdAld at 10 µg/ml each for 5 days and 18 hours prior to termination of experiment 50 µl of XTT was added and the absorbance was read at 480 nm taking 650 nm as reference wavelength. Proliferation was represented as mean OD of stimulated culture - mean OD of unstimulated control. Each bar represents the pooled data (mean ± S.D. value) of 6 individuals and the data represent the means of triplicate wells ± the S.D. Nitric oxide production (µM) by (B) peritoneal macrophages of hamsters and (C) J774 A.1 macrophage cell line. The peritoneal macrophages as well as J774 A.1 cells were primed with the supernatants of stimulated lymphocytes (3 days with mitogen and 5 days with antigens) of normal, infected and cured hamsters in response to rLdEno, rLdAld, SLD and LPS respectively at a concentration of 10 µg/ml each. The estimation of NO production was done using Griess reagent in supernatants collected from macrophage cultures 24 h after incubation and the absorbance of the reaction product was measured at 540 nm. Significance values indicate the difference between the SLD and rLdEno/rLdAld stimulation (*, *p*<0.05; **, *p*<0.01; and ***, *p*<0.001).

The cellular responses of lymph node cells of cured hamsters were assessed by Lymphocyte proliferation test (LTT) against the mitogen ConcavanalinA (ConA) as well as SLD and rLdEno/LdAld at a predetermined concentration of 10 µg/ml. The responses were compared with that of normal as well as *L. donovani* infected groups that served as controls. The normal control as well as cured *Leishmania* infected groups showed significantly higher proliferative responses against Con A, but there were lower mitogenic responses in the cells from *L. donovani*-infected group (Fig. 2 A). The proliferative response of lymphocytes against rLdEno showed significantly higher stimulation in cured/normal hamsters (mean O.D. 2.175±0.495 and 0.933±0.059) than infected control (mean O.D 0.721±0.037). The difference was statistically significant (*p*<0.05). On the other hand, rLdAld showed considerably better response than rLdEno in case of cured hamsters (mean O.D. 2.52±0.80) as compared to the infected ones (mean O.D. 0.97±0.36; *p*<0.001). Another parameter of cellular response is NO-mediated macrophage effector mechanism. The production of NO in peritoneal macrophages of cured hamsters was studied after 24 h of incubation in the presence of rLdEno, rLdAld and SLD whereas LPS stimulated and unstimulated cells served as positive and negative controls respectively. NO production was recorded to be higher (∼2 fold) against rLdEno (28.50±5.41 µM) as well as rLdAld (21.04±3.36 µM) as compared to SLD (13.93±2.11 µM) (Fig. 2 B).

### Lymphoproliferative and Cytokine (IFN-γ, IL-12p40 and IL-10) Responses with PBMCs of Cured/endemic Leishmania Patients in Response to rLdEno/rLdAld

**Figure 3:**
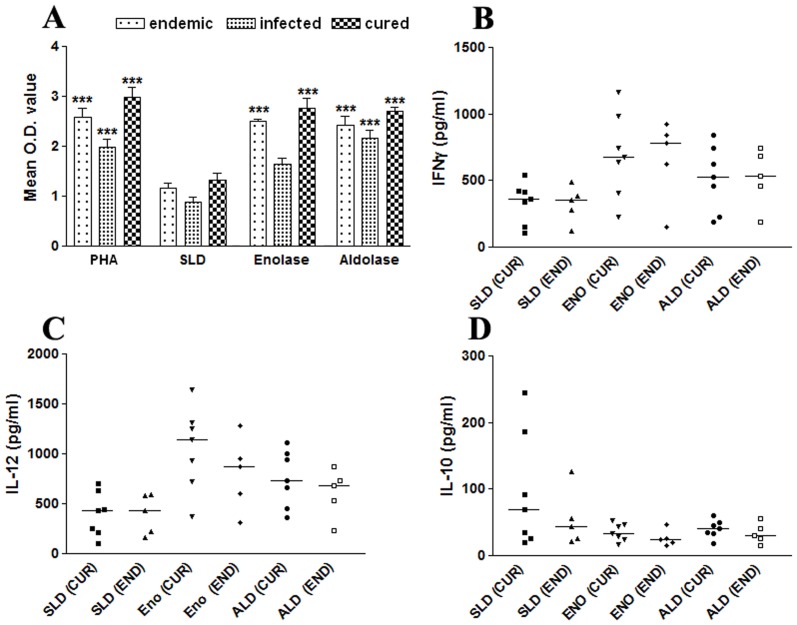
LTT response (A) in PBMCs of endemic, infected and cured patients’ stimulated by SLD, rLdEno and rLdAld at 10 µg/ml concentration for 5 days and 18 hours prior to termination of experiment 50 µl of XTT was added and the absorbance was read at 480 nm taking 650 nm as reference wavelength. Proliferation was represented as mean OD of stimulated culture - mean OD of unstimulated control. Each bar represents the pooled data (mean ± S.D. value) of 6 individuals and the data represent the means of triplicate wells ± the S.D. Cytokine production (B) IFN-γ, (C) IL-12 and (D) IL-10, in stimulated PBMCs from individuals of cured VL patients (CUR; n = 7) and endemic controls (END; n = 5) in response to rLdEno, rLdAld and SLD antigens, each data point represents one individual. The X axis refers to groups of individuals (CUR and END) and the Y-axis corresponds to the values of respective cytokine as concentrations in pg/ml. The mean concentration of cytokine for each group is indicated by the horizontal bars. Cytokine production was tested in triplicate in two independent experiments and the results were comparable. Significance values indicate the difference between the SLD and rLdEno/rLdAld stimulation (*, *p*<0.05; **, *p*<0.01; and ***, *p*<0.001).

The cellular responses (LTT and cytokine levels) of rLdEno/rLdAld were further validated in PBMCs of cured patients, endemic and non-endemic controls and *L. donovani*-infected donors. The proliferative responses in endemic control and cured patients were relatively higher against PHA with mean O.D. values 2.59±0.438 and 2.98±0.479, respectively as compared to unstimulated control. rLdEno showed optimum response in cured (2.76±0.51) as well as endemic contacts (2.50±0.12) which was greater than SLD (1.32±0.34 and 1.17±0.22) (Fig. 3 A). Similar response was observed upon stimulation with rLdAld. The Th1/Th2 stimulatory potential of rLdEno was further assessed by measuring the cytokine levels viz. IFN-γ and IL-12p40 as well as IL-10 in PBMCs from cured patients as well as in endemic contacts. Individual donors in each study group were found to elicit different cellular responses. Optimum stimulation of IFN-γ and IL-12p40 responses was noticed against rLdEno in all the cured patients and endemic contacts (*p*<0.01). The median level of IFN-γ and IL-12p40 in cured patients was to the tune of 690.59 pg/ml (ranging from 224.01 to 1163.06 pg/ml) and 1053.54 pg/ml (ranging from 367.68 to 1643.60 pg/ml), respectively and in endemic contacts it was to the tune of 664.57 pg/ml (ranging from 151.01 to 921.80 pg/ml) and 804.18 pg/ml (ranging from 315.79 to 1286.36 pg/ml), respectively (Fig. 3 B and C). However, the IFN-γ response upon stimulation with rLdAld in cured and endemic contacts was 515.10 pg/ml (ranging from 188.03–843.28 pg/ml) and 522.24 pg/ml (ranging from 189.15–742.53 pg/ml) respectively whereas IL-12 response was 752.25 pg/ml (ranging from 361.09–1113.32 pg/ml) in cured subjects and 610.64 pg/ml (ranging from 235.43–872.43 pg/ml) in endemic contacts. Moreover, no detectable amount of IFN-γ and IL-12p40 level was observed with the PBMCs of the *L. donovani*-infected patients and healthy individuals against rLdEno as well as rLdAld. On the contrary, the level of IL-10 cytokine against rLdEno stimulation (Fig. 3 D) was quiet low in cured (35.45 pg/ml ranging from 17.12 to 53.12 pg/ml) as well as in endemic contacts (25.96 pg/ml ranging from 15.10 to 46.49 pg/ml). Similar response of IL-10 production was observed in case of rLdAld stimulation. In case of all the five infected patients the IL-10 level in response to rLdEno and rLdAld was significant and found to be increased (158.63 pg/ml (rLdEno) and 219.12 pg/ml (rLdAld); *p*<0.01) as compared to cured/endemic as well as healthy (2.6–9.2 pg/ml) individuals (data not shown) but was, however, inferior to that of SLD (349.6 pg/ml, ranging from 190.56–540.41 pg/ml) (Fig. 3 D).

### Vaccination with rLdEno/rLdAld +BCG Induced Optimum Protection against *L. donovani* Challenges

**Figure 4:**
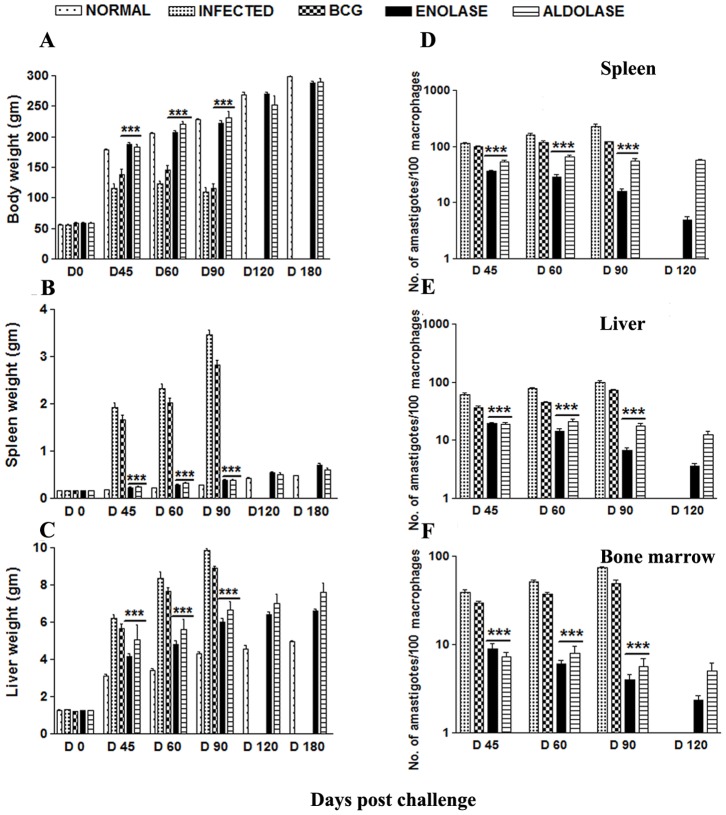
Clinical outcomes following *L. donovani* challenge in hamsters immunized with rLdEno+BCG and rLdAld+BCG. (A) Body weight, (B) spleen weight, and (C) liver weight in grams; on day 21 after the booster, the hamsters of infected, BCG alone and vaccinated groups were challenged intracardially with 107 metacyclic promastigotes of *L. donovani*. On days 45, 60, 90 and 120 p.c., 3–4 animals from each group were sacrificed and weights of body, liver and spleen were recorded. Parasite burden (no. of amastigotes per 100 cell nuclei) in the (D) spleen, (E) liver, and (F) bone marrow on days 45, 60, 90, and 120 p.c. Significance values indicate the difference between the vaccinated groups and infected group (*, *p*<0.05; **, *p*<0.01; and ***, *p*<0.001). Data represent mean values standard errors (SE) at the designated time points and are representative of two independent experiments with similar results.

The hamsters vaccinated with rLdEno/rLdAld+BCG gained substantial weight as compared to those hamsters which were immunized with BCG alone as well as to unimmunized infected animals, when kept simultaneously for the same time period, i.e. on days 45, 60, 90 and 120 post challenge (p.c) (Fig. 4 A–C). A significant reduction in parasite load [∼90%, *p*<0.001 (rLdEno); ∼65%, *p*<0.001 (rLdAld)] was observed in spleen, liver and bone marrow of rLdEno/rLdAld+BCG vaccinated animals on day 90 p.c (Fig. 4 D–F). All the vaccinated hamsters survived longer after the lethal challenge of *L. donovani* and remained healthy until the termination of the experiment at 5–8 months post-infection. On the other hand, there was progressive increase in parasite load in the hamsters immunized with BCG alone as well as unimmunized ones and they succumbed to virulent *L. donovani* challenge within 2–3 months.

### Vaccination with rLdEno/rLdAld+BCG Stimulate Substantial Delayed type Hypersensitivity, Mitogenic and Cellular Responses in Hamsters against *L. donovani* Challenges

**Figure 5:**
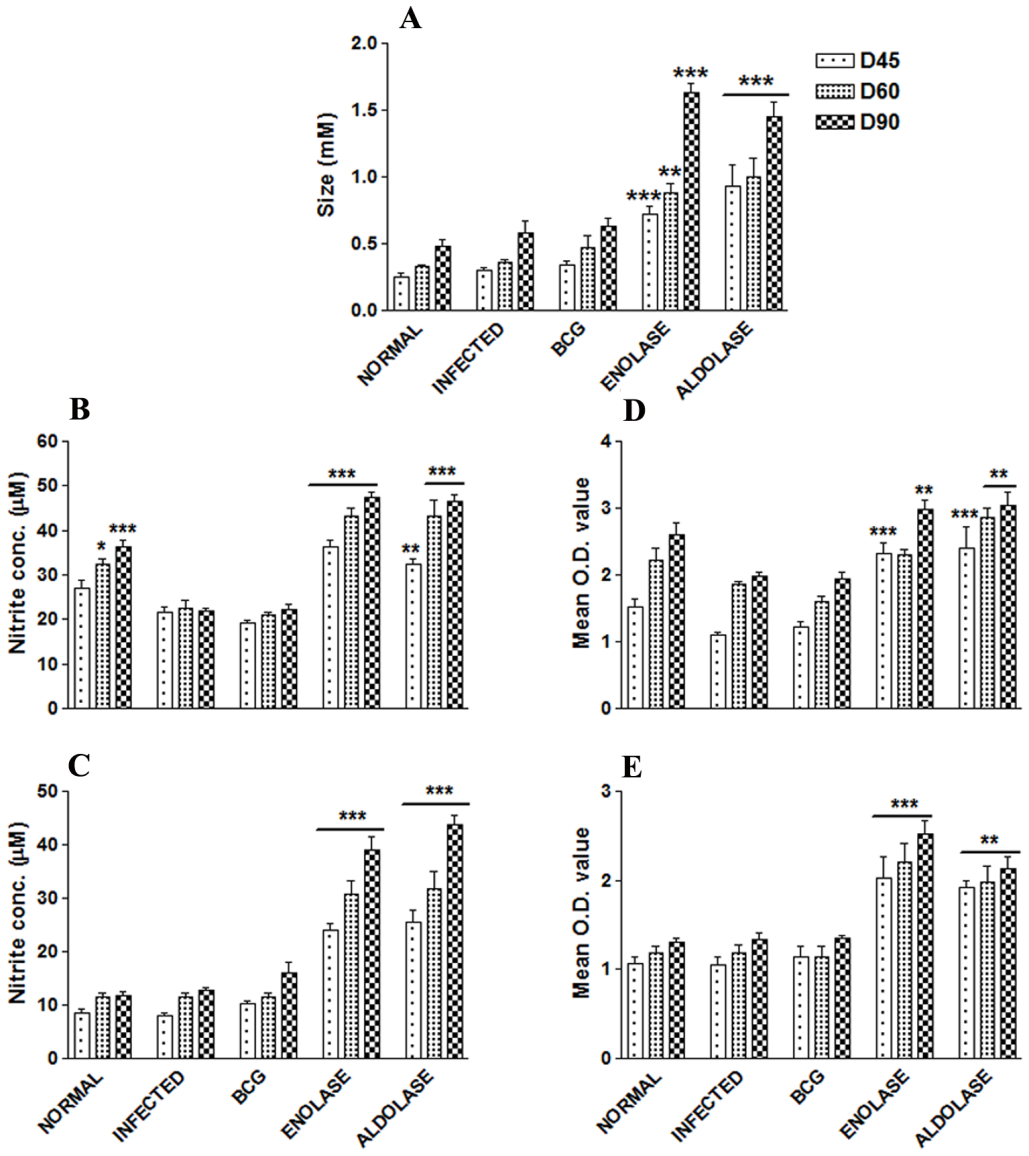
DTH response (mM) to SLD as footpad swelling on days 45, 60 and 90 p.c. (A); NO production (µM) to LPS (B) and rLdEno/rLdAld (C) in the naïve macrophages co-incubated with supernatants of lymphocytes isolated from rLdEno/rLdAld vaccinated hamsters in comparison to the BCG vaccinated, infected controls, and uninfected normal hamsters on days 45, 60, and 90 p.c. The level of nitrite in the culture supernatants was assessed was assessed by Griess reagent. LTT response to ConA (D) and rLdEno/rLdAld (E) stimulation in vaccinated hamsters, BCG vaccinated, infected controls and uninfected normal hamsters. Results are means ± SE for 3–4 hamsters per group done in duplicate and are representative of two independent experiments with similar results. Significance values indicate the difference between the vaccinated group and infected group (*, *p*<0.05; **, *p*<0.01; and ***, *p*<0.001).

The cellular response generated following immunization with rLdEno/rLAld+BCG in hamsters and challenged with *L. donovani* was characterized. There was a significantly stronger level of DTH response in vaccinated (both rLdEno/rLdAld+BCG) hamsters as compared to the other control groups which gradually increased after 2 and 3 months (*p<*0.001) (Fig. 5 A). LTT response induced by Con A in vaccinated animals was similar to that observed in normal ones throughout the entire p.c. period (days 45, 60 and 90 p.c.) whereas it was lower in other control groups (Fig. 5 D). In antigen-specific re-stimulation assays, there was noteworthy (*p*<0.001) stimulatory response in the cells of hamsters vaccinated with rLdEno+BCG while a slightly lower response in case of hamsters vaccinated with rLdAld+BCG (*p*<0.01) (Fig. 5 E). On the other hand, there was lesser proliferative response in animals vaccinated with BCG alone as well as unvaccinated infected control.

Since, generation of NO after macrophage activation by IFN-γ is an important factor in controlling leishmaniasis, [50] the NO content in lymphocytes was measured in all the vaccinated hamsters after *L. donovani* challenge. Macrophages isolated from naïve hamsters, when incubated with LPS and supernatants of stimulated lymphocytes from rLdEno+BCG vaccinated hamsters, produced significant amount of NO (23.96±2.21 µM; *p*<0.001) than the other control groups (8.2–10.2 µM values) on day 45 p.c (Fig. 5 B,C). Further increase in NO level was observed by days 60 and 90 p.c. (Fig. 5 B). Similar results were observed with hamsters vaccinated with rLdAld+BCG. (25.47±4.16 µM; *p*<0.001).

### Vaccination with rLdEno/rLdAld +BCG Elicits Prominent Th1-type Cytokine Responses, as Assessed by RT-PCR, in Hamsters against *L. donovani* Challenges

**Figure 6:**
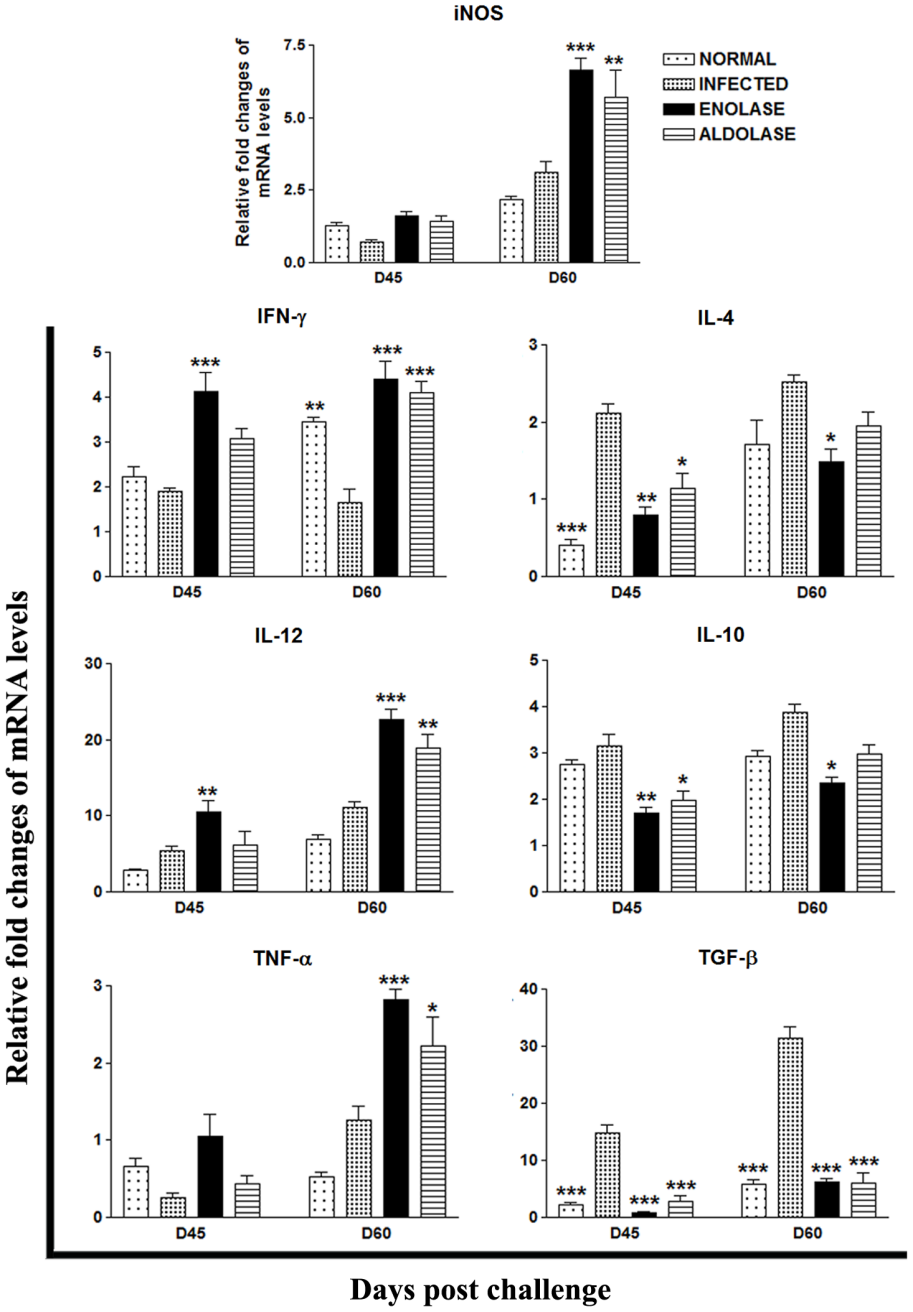
Splenic iNOS and cytokine (Th1/Th2) mRNA expression profile analysis of normal, infected rLdEno+BCG and rLdAld+BCG vaccinated hamsters (n = 3/4) on day 60 p.c. by qRT-PCR. Data are presented as means ± SE and are representative of two independent experiments with similar results. Significance values indicate the difference between the vaccinated group and infected group (*, *p*<0.05; **, *p*<0.01; and ***, *p*<0.001).

The mRNA expression of Th1 and Th2 cytokines viz. IFN-γ, TNF-α, IL-12, TGF-β, IL-4, IL-10 and iNOS in hamsters of rLdEno/rLdAld+BCG vaccinated group was evaluated on days 45 and 60 p.c. and compared to animals infected with *L. donovani* as well as normal control groups. An increase in the expression levels of Th1 type of cytokines was observed in hamsters vaccinated with rLdEno+BCG on days 45 and 60 wherein a significantly high level of IFN-γ (∼2 folds; *p*<0.001) was noticed at both time points as compared to the infected controls (Fig. 6). On the other hand the expression of IFN-γ in hamsters vaccinated with rLdAld+BCG was slightly lesser as compared to the rLdEno+BCG vaccinated hamsters and significant only at day 60 (*p*<0.001). A moderate increase in expression levels of iNOS and TNF-α mRNA transcripts was observed by hamsters vaccinated with rLdEno+BCG on day 45 p.c. which further increased significantly on day 60 p.c. by ≥3 folds (*p*<0.001), while the hamsters vaccinated with rLdAld+BCG generated a similar but lower response. In infected animals mRNA transcripts of TGF-β, IL-4 and IL-10 were highly up regulated whereas the expression of mRNA for IL-12 and IFN-γ was found to be down regulated. In case of Th1 deactivating cytokines an extreme down-regulation in the expression levels of TGF-β (2–4 folds; *p<*0.001) was observed in the both the vaccinated groups as compared with the infected ones at both time points. On the other hand IL-4 and IL-10 expression was downregulated in both the vaccinated groups on day 45 (*p<*0.01 in case of rLdEno+BCG vaccinated and *p<*0.05 in case of rLdAld+BCG vaccinated) and followed by significant downregulation on day 60 p.c. in case of rLdEno+BCG vaccinated hamsters.

### IgG and its Isotypes IgG1 and IgG2 Antibody Responses in Vaccinated Hamsters

**Figure 7:**
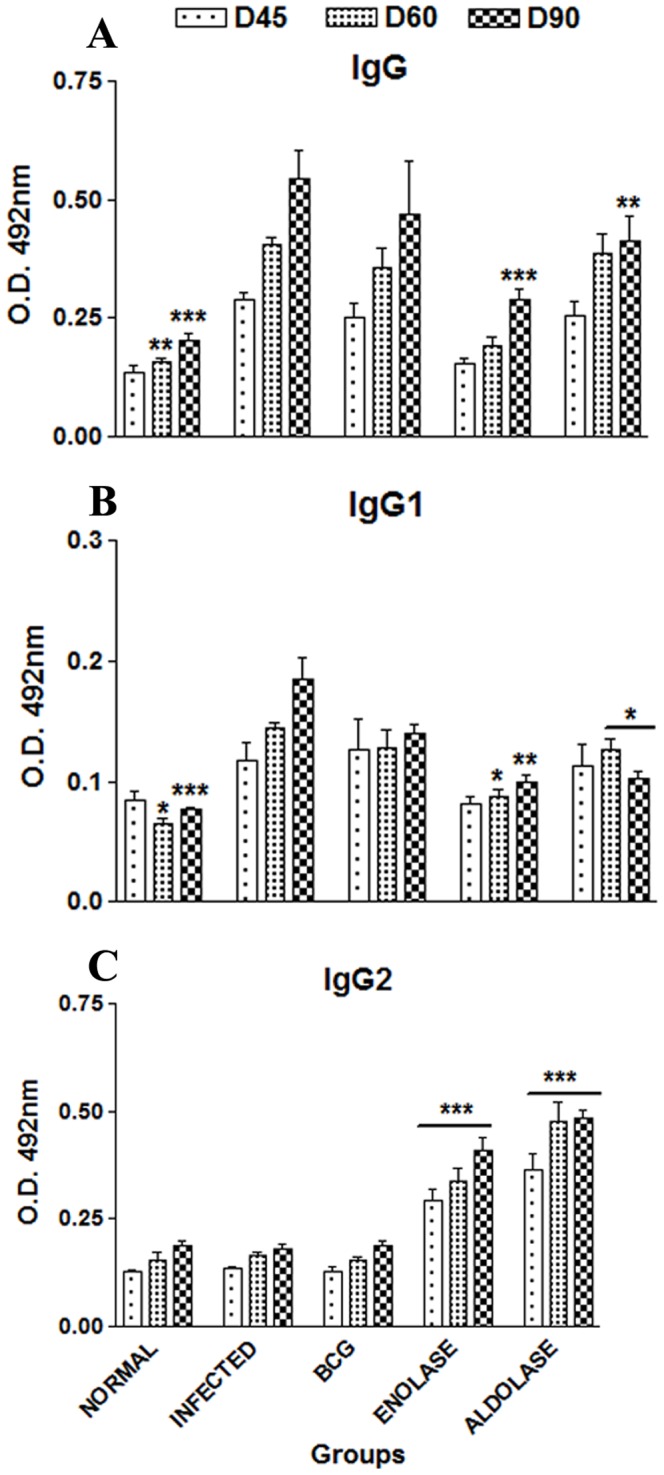
Antibody response in vaccinated hamsters. (A) IgG and its isotypes (B) IgG1and (C) IgG2 in rLdEno-vaccinated hamsters in comparison to the unimmunized infected hamsters on days 45, 60, and 90 p.c. Serum samples were collected from different groups of hamsters at designated time points and assayed for rLdEno/rLdAld- specific IgG, IgG1, and IgG2 levels by ELISA. Data are presented as the absorbance at 492 nm and are means ± SE for 3–4 hamsters per group in triplicate wells. Data are representative of two independent experiments with similar results. Significance values indicate the difference between the vaccinated and infected group (*, *p*<0.05; **, *p*<0.01; and ***, *p*<0.001).

The hamsters vaccinated with rLdEno+BCG exhibited significantly higher levels of IgG2 antibody (O.D. value 0.3427±0.012; *p*<0.001) which was remarkably different from the infected control groups (O.D. value 0.180±0.021) (Fig. 7 C). Similar results were observed in case of rLdAld+BCG vaccinated hamsters. In contrast, decreased levels of IgG and IgG1 in vaccinated hamsters (O.D. values 0.225±0.036 for IgG and 0.099±0.011 for IgG1) were observed as compared to the infected controls (O.D. values 0.4076±0.03 for IgG and 0.166±0.016 for IgG1) (Fig. 7 A and B).

### T-Cell Epitope Prediction

We predicted 9-mer and 10-mer promiscous peptides for MHC-1 and MHC-II respectively. Promiscous epitopes having percentile score ≤45 were selected. BlastP search was performed to identify the identity with human protein if any and peptides showing high identity (>85) were not considered. Finally for MHC-I alleles six potential promiscus epitopes were listed from LdEno and five from LdAld (Table 2). Similarly for MHC-II alleles six promiscous peptides from both LdAld and LdEno were listed (Table 3).

**Table 2:** MHC-I predicted epitopes from LdAld and LdEno with positional information.

S.No	LdEno Epitope sequence	Percentile score				
		HLA-A*01∶01	HLA-A*24∶02	HLA-A*33∶03	HLA-A*33∶06	HLA-A*02∶11
1.	AILGCSMAI	39.45	12.2	40	40	4.1
2.	LTVTNVERV	2.8	26.85	21	21	3.7
3.	MGSEVYHAL	10.3	17.95	12	12	6.9
4.	YIAGLAGTK	22.85	27.45	12	12	24.6
5.	ITMALAGKA	8.75	34.85	15	15	16.2
6.	MALAGKAQI	43.8	16.4	12	12	11.4
**S.No**	**LdAld Epitope sequence**	**Percentile score**				
		HLA-A*01∶01	HLA-A*24∶02	HLA-A*33∶03	HLA-A*33∶06	HLA-A*02∶11
1.	GVILHEETV	40.8	38.1	42	42	8.35
2.	AVRFNAETL	44.75	24.2	32	32	17.45
3.	FNAETLARY	4.15	46.65	24	24	44.05
4.	AILSQISGL	39.75	35.5	39	39	2.9
5.	ILSQISGLV	8.65	15.2	28	28	1.65

Note: For HLA-A*01∶01, HLA-A*24∶02 and HLA-A*02∶11 percentile score was calculated using ANN and SMM. For HLA-A*33∶03 and HLA-A*33∶06 percentile score was based on NetMHCpan.

**Table 3:** MHC-II predicted epitopes from LdAld and LdEno with positional information.

No.		Epitope sequence (LdAld)	Position
1.		TLARYAILSQISGLV	179–193
2.		LARYAILSQISGLVP	180–194
3.		LDGYVKRASAYYKKG	137–151
4.		MSRVTIFQSQLPACN	1–15
5.		YTVMTLARTMPAMLP	261–275
6.		VMTLARTMPAMLPGV	263–277
**No.**	**Epitope sequence (LdEno)**		**Position**
1.	MPIRKVYAREVLDSR		1–15
2.	VLPFQEFMIAPTKAT		160–174
3.	HALKVIIKSKYGQDA		188–202
4.	LYRYIAGLAGTKDIR		128–142
5.	SEVYHALKVIIKSKY		184–198
6.	ACNSLLLKINQIGTI		336–350

## Discussion

In VL, Th1 immune responses play an important role in controlling the disease hence, T-cell stimulatory antigens are thought to be good vaccine targets. Consequently, search of such antigens was made which could elicit cellular responses in PBMCs/lymphocytes of cured *Leishmania* patients/hamsters. In earlier studies from our laboratory, while carrying out classical activity based fractionation and sub-fractionation of soluble *L. donovani,* we have observed that the sub-fraction ranging from 89.9 to 97.1 kDa elicited significant Th1 stimulatory response in PBMCs/lymphocytes of cured *Leishmania* patients/hamsters. The proteomic characterization of this sub-fraction revealed a number of immunogenic proteins [6] and Enolase as well Aldolase were identified amongst them. The presence of Enolase and aldolase in higher molecular weight range in proteomic studies, in contrast to its observed molecular mass could be attributed to the post-translational modifications which are widely prevalent in *Leishmania.* Both Enolase and Aldolase are vital protein/enzyme of glycolytic pathway and may be exploited as both vaccine as well as drug targets [51], [52]. *Streptococus suis* enolase (SsEno), shown to possess highly conserved epitopes, is expressed at the surface and identified as an important antigenic protein that contributes to the virulence and therefore might be an attractive vaccine candidate against *S. suis* infections [24], [25], [53]. Enolase has also been described as one of the immunomodulatory proteins of *S. Sorbinus* [25]. In case of *Candida albicans*, enolase promotes host endothelial invasion by binding to plasmin(ogen) and plays a protective role in *C. albicans* infection [20], [21], [22]. It has also been evaluated as vaccine candidate in case of *Ascaris suum* and *Echinococcus granulosus* [54], [55]. Similarly, FBA has also been found to be protective in *Onchocerca volvulus*, *Fasciola hepatica* and *Schistosoma mansoni* [7], [9], [10]. As these proteins have not been evaluated as vaccine candidates in case of VL, it was pertinent to assess these proteins for their immunogenicity and prophylactic potential against VL. For this, we first cloned, overexpressed and purified the proteins to homogeneity. The antibody generated against rLdEno and rLdAld in rabbit were observed to be specific to *Leishmania* and detected single band against whole cell lysate and SLD of *Leishmania* promastigotes. Both the proteins were purified in native conditions and were enzymatically active as determined by studying their kinetic parameters which were similar to that observed earlier [31], [56]. *In-silico* sequence and structural comparison of LdAld and LdEno with human counterpart exposed key differences in the active site of both glycolytic enzymes. These differences may provide platform to design specific inhibitors.

A major factor contributing to healing in leishmaniasis is the development of strong cell mediated immune response [57]. The measures of cell-mediated immunity are *Leishmania* specific lymphoproliferation and the stimulation of T-cells to produce macrophage activating factor, including IFN-γ which in turn activate macrophages to kill the intracellular parasites. It has been reported earlier that upon stimulation with SLD and its subfractions, a T-cell response develops in cells (lymphocytes) from exposed or individuals infected with *Leishmania* and cured using anti-leishmanials [27], [58]. Further, it is well established that recovery from *Leishmania* infection, relies on induction of Th1 response [59], [60] with production of IFN-γ, IL-12 and enhanced expression of nitric oxide synthase [61]. In the absence of cytokine reagents for hamsters, nitric oxide assay was used to indirectly estimate the IFN-γ response, as NO is up regulated by IFN-γ. Therefore, when assessed for their immunogenicity *in vitro*, rLdAld gave significantly higher LTT response than rLdEno while the NO response against cured hamsters in comparison to normal and infected ones was almost 2–3 times enhanced than that observed in case of SLD and was better in case of rLdEno than rLdAld. The generation of NO supports the up-regulation of iNOS by Th1 cell-associated cytokines. The characterization of the cellular immune response was first performed in cured *Leishmania* infected hamsters and then the responses of both the proteins were validated in endemic non immune donors (household contacts without any clinical symptoms) and in immune patients of VL that were cured with amphotericin B following the similar protocol as described earlier [27], [62], who have demonstrated the development of a good T-cell response, when cells from these individuals are stimulated with different fractions of *Leishmania* antigen. This observation was authenticated in endemic control and cured patients of VL wherein analogous results were observed, i.e. proliferation of lymphocytes *in vitro* and the release of very high amount of Th1-type cytokines viz. IFN-γ and IL-12p40 in response to rLdEno as well as rLdAld and compared with SLD.

The induction of lymphocyte proliferation and IFN-γ production by some of the recombinant antigens viz. gp63 in subjects cured of visceral form and in patients with cutaneous or mucosal leishmaniasis has been shown by different groups [63], [64]. These observations can be correlated with the well documented fact that IFN-γ induces production of NO in phagocytic cells (principally macrophages) harbouring leishmania parasite, thereby killing them. The capacity to produce IFN-γ after antigenic stimulation of immune lymphocytes may be an important indicator of effective cell-mediated immunity. Thus, the similar cellular responses to rLdEno as well as rLdAld in cured hamsters as well as in cured patients/endemic controls of VL indicate that the results so obtained with the hamster could be translated into humans. The limitations of this *in vitro* study based on a convenience human sampling, may not perhaps allow drawing solid conclusions regarding the immunogenicity of rLdEno as well as rLdAld.

We further evaluated the prophylactic potential of rLdEno as well as rLdAld along with BCG in hamsters by challenging them with lethal dose of *Leishmania*. BCG had been used as an adjuvant [65], [66] with several immunizing agents [65], [66], [67] since it has been reported that it activates macrophages inducing NO [68], [69] and elicits long lasting cellular and humoral immune responses [70]. Significant reduction in parasite load (∼90%, *p*<0.001) was noticed in rLdEno vaccinated hamsters and was supported by their longer survival period (>180 days p.c.) as compared to unvaccinated infected ones (<90 days p.c.), while rLdAld provided only partial protection (∼65%, *p*<0.001). A positive correlation of parasite loads with splenomegaly and hepatomegaly was observed among the experimental and control groups.

Among the several parameters of CMI, one measure is *Leishmania*- specific LTT related to T cell stimulation with mitogens and antigen *in vitro*, which almost always accompanies control of parasite growth and healing in humans and animals [3], [67], [71]. Since previous reports described that T cell proliferation is impaired during active VL [50], we explored rLdEno as well as rLdAld-induced T cell proliferation in hamsters vaccinated with rLdEno+BCG and rLdAld+BCG after *L. donovani* challenge at different time points of study. Significant LTT response (∼2.5 folds) in rLdEno+BCG vaccinated hamsters and ∼2 folds in rLdAld+BCG vaccinated hamsters was observed on days 45, 60 and 90 p.c. as compared to the other experimental as well as control groups. However, LTT itself is not the only primary effector mechanism of immunity, but rather the production of NO, upon stimulation of *Leishmania*-specific T cells, activates the macrophages to kill the intracellular parasites [72]. In this study also, there was a gradual increase in NO production (∼2–3 folds) in the supernatant of macrophages co-stimulated with supernatant of rLdEno as well as rLdAld-stimulated lymphocytes from vaccinated hamsters which also support the view regarding the up-regulation of iNOS by Th1 cell associated cytokines. Successful vaccination of humans and animals is often related to antigen induced DTH responses *in vivo* [73] which is characterized by activation and recruitment of predominantly T cells and macrophages at the site of intradermal injection in previously sensitized host [74], [75]. DTH has been shown to be absolutely dependent on the presence of memory T-cells. Both the CD4+ and CD8+ fractions of cells have been shown to modulate an immune response. Contemporary debate regarding the reaction is focused on the role of the Th1 and Th2 cells [76]. It has been postulated that the Th1 cell is the “inducer” of a DTH response since it secretes IFN-γ, a potent stimulator of macrophages, while the Th2 cell is either not involved or act as a downregulator of the cell mediated immune response. Notably, the vaccinated hamsters’ elicited strongest DTH reaction among other experimental groups suggesting a correlation between CMI responses and immunity to infection. Herein too, a significant DTH response was observed in the hamsters vaccinated with rLdEno+BCG and rLdAld+BCG.

It is well established that the cytokine milieu at the initiation of infection is critical in determining disease outcome [77], [78]. So to understand the interplay between the disease healing inflammatory cytokines IFN-γ and IL-12 and disease associated cytokines IL-10 and IL-4, the expression of these cytokines as well as the level of iNOS transcript was investigated by qRT-PCR. As the reagents for cytokine estimation in hamsters is commercially unavailable, herein quantitative real time PCR was used for assessing the expression of cytokine mRNAs in the experimental groups. The immune response generated thereof in vaccinated hamsters was clearly associated with a Th1-type cytokine response predominantly, as indicated by the upregulation of iNOS, IFN-γ, and IL-12, along with simultaneous downregulation of TGF-β, IL- 4, and IL-10. Transcripts of IFN-γ and TNF-α, often reported to act in concert to activate iNOS for the production of NO [79], showed manifold increase in all the immunized groups of hamsters. It is also suggested that TNF-α is one of the primary agents to stimulate macrophage to produce NO [80]. Our results are in consistence with the studies of Bhowmick *et al* [5] in which the control of disease progression and parasite burden in vaccinated mice was associated with augmentation of antigen-specific IFN-γ production and down-regulation of IL-4, demonstrating a Th1 bias. High levels of IL-4 and IL-10 that has been observed in unimmunized infected control animals supported the view that marked up-regulation of these two cytokines is accompanied by susceptibility, disease progression and depressed Th1 type of cell mediated immunity [81], [82], [83]. The presence of IL-4, IL-10 and TGF-β in infected hamsters are reported to be the major immunosuppressive cytokines in experimental and human VL [3], [43], [84], [85], [86], [87]. The studies of Lehmann *et al* [88] also established that in VL a Th1 dominated immune response is protective and, furthermore, the capacity to produce IFN- γ rather than the presence of IL-4 determines the efficacy of the immune response in susceptible mice. In addition, a marked decrease in the expression of IL-10 in vaccinated hamsters, which was not observed in positive control, may be due to an inhibitory effect of IFN-γ in the expression of this cytokine [89]. TGF-β, a pleiotropic cytokine with diverse functions, is known to be expressed at a moderate level even in normal hamsters [3], [43], [90]. TGF-β is also known to inhibit the activities of immune cells and was found to be down-regulated in vaccinated hamsters compared with the infected controls. Unlike the findings of [90], where they could not detect IL- 4 transcripts at all in splenocytes of >90% of the infected hamsters, it was quite evident in this study. Further, there was apparent down regulation of IL-4 expression in all the immunized hamsters throughout the experiment.

Moreover, active VL is also associated with the production of high levels of the *Leishmania* specific antibody which is observed before detection of parasite-specific T cell response [91]. The progressive elevation of the anti-*Leishmania* IgG and IgG1 in all the experimental groups except the vaccinated groups (rLdEno+BCG and rLdAld+BCG), compared with the level of IgG2, which was significantly and consistently prominent (∼1.5–2 folds) in the vaccinated groups, suggested that protection against leishmaniasis is induced by a strong T cell response. These observations further support the views that protection against leishmaniasis is induced by a strong T-cell response and almost undetectable amounts of antibodies [92], [93]. This pattern was also seen in clinical as well as experimental VL [94], [95]. To further understand the molecular basis of the immune response elicited by these proteins, epitope prediction for both MHC-I and MHC-II potential promiscous epitopes from LdAld and LdEno sequences were carried out. We focused on 9-mer long peptides since HLA class I binds 9-mers more frequently than 8, 10 or 11-mer peptides. Both LdEno and LdAld had 6 epitopes for MHC class II epitopes while LdEno had 6 and LdAld had 5 epitopes for MHC Class I.

To our knowledge, this is the first report regarding the potentiality of enolase –a glycolytic protein, as possible vaccine candidate against VL. In addition, *in-silico* analysis of LdAld and LdEno has suggested that LdAld and LdEno both can be potential vaccine candidates.

## Supporting Information

Figure S1 **Cloning, expression, purification and western blot analysis of enolase.** (A) Specific PCR of enolase, Lane M: 100 bp ladder, lanes 1 & 2: amplified PCR product at 1290 bp; (B) Clone confirmation in pTZ57R/T, Lane M: 100 bp ladder, lane 1: *BamHI*-*EcoRI* digested pTZ57R/T+Enolase, lane 2: Undigested plasmid; (C) Clone confirmation in pET28a, Lane M: 100 bp ladder, lane 1: *BamHI-EcoRI* digested pET28a+Enolase, lane 2: Undigested plasmid; (D) Purification of rLdEno: Lane M: Molecular weight marker, lane 1: WCL of pET28a+Enolase before IPTG induction, lane 2: Supernatant of induced (0.1 mM IPTG and 1 hour) and sonicated lysate of pET28a+Enolase, lane 3: Flowthrough of induced and sonicated lysate after equilibration in Ni-NTA column, lane 4: Purification of recombinant Enolase, lane 5–9: Eluted purified rLdEno; (E) Western blot using rabbit anti-enolase sera (dilution 1∶10,000). Lane M: Molecular weight marker, lane 1: Whole cell lysate of *L. donovani*; lane 2: Soluble *L. donovani* antigen. The samples were run on 12% SDS-PAGE and transferred onto nitrocellulose membrane. The preimmune sera of the rabbit did not reacted with the protein (data not shown).
(TIF)

Figure S2 **Cloning, expression, purification and western blot analysis of Aldolase.** **(A)** Specific PCR of Aldolase, Lane M: 1 kb ladder, lane 1: amplified PCR product at 1125 bp; **(B)** Clone confirmation in pTZ57R/T, Lane M: 100 bp ladder, lane 1: *BamHI*-*EcoRI* digested pTZ57R/T+Aldolase, lane 2: Undigested plasmid; **(C)** Clone confirmation in pET28a, Lane M: 1 kb ladder, lane 1: *Bam HI-EcoRI* digested pET28a+Aldolase, lane 3: Undigested plasmid; **(D)** Purification of rLdAld: Lane M: Molecular weight marker, lane 1: WCL of pET28a+Aldolase before IPTG induction, lane 2: WCL of pET28a+Aldolase after IPTG induction (1 mM IPTG and 4 hours), lane 3: Supernatant of induced and sonicated lysate of pET28a+Aldolase, lane 4: Flowthrough of induced and sonicated lysate after equilibration in Ni-NTA column, lane 5 & 6: Wash fractions 1 & 3, lanes 7–9: Eluted purified rLdAld. **(E)** Western blot using anti-Aldolase antibody. Lane M: Molecular weight marker; lane 1: WCL of pET28a+Aldolase before IPTG induction, lane 2: WCL of pET28a+Aldolase after IPTG induction, lane 3: Purified rLdAld, lane 4: Soluble *L. donovani* antigen.
(TIF)

Figure S3 **Multiple sequence alignment of enolase sequences of** ***Leishmania donavani*** **(LdEno; ACE74540.1),** ***Trypansoma brucei*** **(TbEno; Q9NDH8),** ***Saccharomyces cerevisiae*** **(ScEno;** P00924**), Human (HsEnoalpha; P06733, HsEnobeta; P13929 and HsEnogamma; P09104).** Fully conserved active site residues are shown by black rectangular dots, while residues near to active site which differs from Human enolase, are shown in green rectangular dots. Alignment image was produced by ESPript (www.espript.ibcp.fr/ESPript/ESPript/) after submitting aligned sequences generated by ClustalW (www.ebi.ac.uk/Tools/msa/clustalw2/).
(TIF)

Figure S4 **Multiple sequence alignment of aldolase sequences of** ***Leishmania donovani*** **(LdEno; ACT67434.1),** ***Leishmania mexicana*** **(LmAld; CAB55315.1** ***) Trypansoma cruzi*** **(TbEno; XP_809370.1) and** ***Homo sapiens*** **(HsAldA1; NP_001121089.1, HsAldB; NP_000026.2 and HsAldC; NP_005156.1).** Fully conserved active site residues are shown by black rectangular dots, while residues near to active site which differs from Human enolase, are shown in green rectangular dots. Alignment image was produced by ESPript (www.espript.ibcp.fr/ESPript/ESPript/) after submitting aligned sequences generated by ClustalW (www.ebi.ac.uk/Tools/msa/clustalw2/).
(TIF)

Figure S5 **Immunofluorescence analysis of the intracellular distribution of enolase (LdEno) and aldolase (LdAld) in** ***L. donovani*** **promastigotes; (A) The images showing a diffusely pattern of LdEno throughout the cell body with a marked exclusion of the nucleus; (B) The images showing localization of LdAld in the discrete granular compartments, presumably the glycosomes, apart from its localization through the flagellar length.** Arrowheads mark the flagellar localization of LdAld. a, immuno-fluorescence images; b, differential interference contrast image; c, nuclei and kinetoplasts labelled with DAPI; d, merged images.
(TIF)
