# Supplementary figures and images for: Characterization of Glycolytic Enzymes - rAldolase and rEnolase of Leishmania donovani, Identified as Th1 Stimulatory Proteins, for Their Immunogenicity and Immunoprophylactic Efficacies against Experimental Visceral Leishmaniasis
Source: PLoS One. 2014 Jan 24;9(1):e86073. doi: 10.1371/journal.pone.0086073 (PMC3901665; doi:10.1371/journal.pone.0086073)

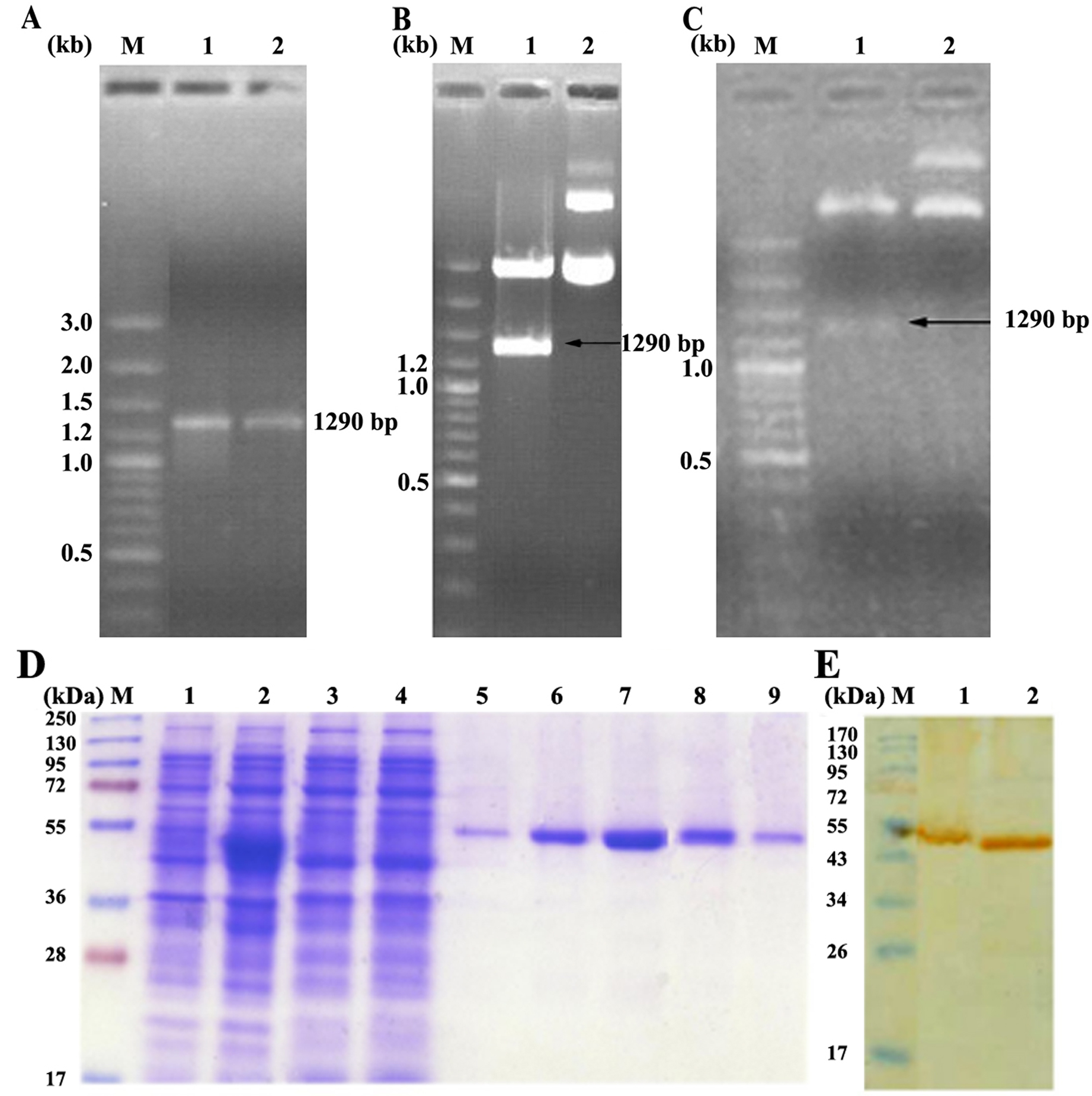

Supplement: Figure S1 — Cloning, expression, purification and western blot analysis of enolase. (A) Specific PCR of enolase, Lane M: 100 bp ladder, lanes 1 & 2: amplified PCR product at 1290 bp; (B) Clone confirmation in pTZ57R/T, Lane M: 100 bp ladder, lane 1: BamHI-EcoRI digested pTZ57R/T+Enolase, lane 2: Undigested plasmid; (C) Clone confirmation in pET28a, Lane M: 100 bp ladder, lane 1: BamHI-EcoRI digested pET28a+Enolase, lane 2: Undigested plasmid; (D) Purification of rLdEno: Lane M: Molecular weight marker, lane 1: WCL of pET28a+Enolase before IPTG induction, lane 2: Supernatant of induced (0.1 mM IPTG and 1 hour) and sonicated lysate of pET28a+Enolase, lane 3: Flowthrough of induced and sonicated lysate after equilibration in Ni-NTA column, lane 4: Purification of recombinant Enolase, lane 5–9: Eluted purified rLdEno; (E) Western blot using rabbit anti-enolase sera (dilution 1∶10,000). Lane M: Molecular weight marker, lane 1: Whole cell lysate of L. donovani; lane 2: Soluble L. donovani antigen. The samples were run on 12% SDS-PAGE and transferred onto nitrocellulose membrane. The preimmune sera of the rabbit did not reacted with the protein (data not shown). (TIF) [file pone.0086073.s001.tif]

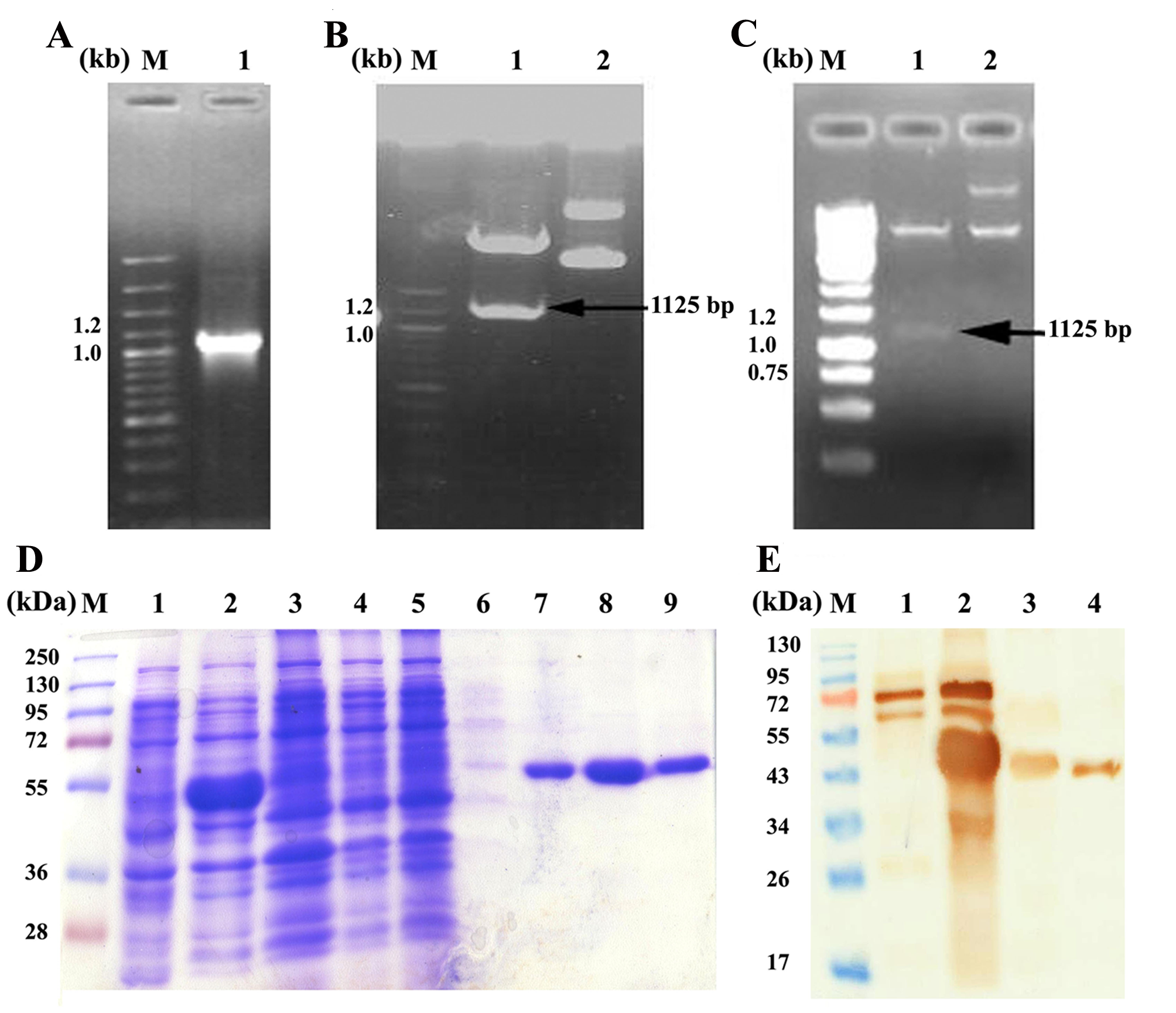

Supplement: Figure S2 — Cloning, expression, purification and western blot analysis of Aldolase. (A) Specific PCR of Aldolase, Lane M: 1 kb ladder, lane 1: amplified PCR product at 1125 bp; (B) Clone confirmation in pTZ57R/T, Lane M: 100 bp ladder, lane 1: BamHI-EcoRI digested pTZ57R/T+Aldolase, lane 2: Undigested plasmid; (C) Clone confirmation in pET28a, Lane M: 1 kb ladder, lane 1: Bam HI-EcoRI digested pET28a+Aldolase, lane 3: Undigested plasmid; (D) Purification of rLdAld: Lane M: Molecular weight marker, lane 1: WCL of pET28a+Aldolase before IPTG induction, lane 2: WCL of pET28a+Aldolase after IPTG induction (1 mM IPTG and 4 hours), lane 3: Supernatant of induced and sonicated lysate of pET28a+Aldolase, lane 4: Flowthrough of induced and sonicated lysate after equilibration in Ni-NTA column, lane 5 & 6: Wash fractions 1 & 3, lanes 7–9: Eluted purified rLdAld. (E) Western blot using anti-Aldolase antibody. Lane M: Molecular weight marker; lane 1: WCL of pET28a+Aldolase before IPTG induction, lane 2: WCL of pET28a+Aldolase after IPTG induction, lane 3: Purified rLdAld, lane 4: Soluble L. donovani antigen. (TIF) [file pone.0086073.s002.tif]

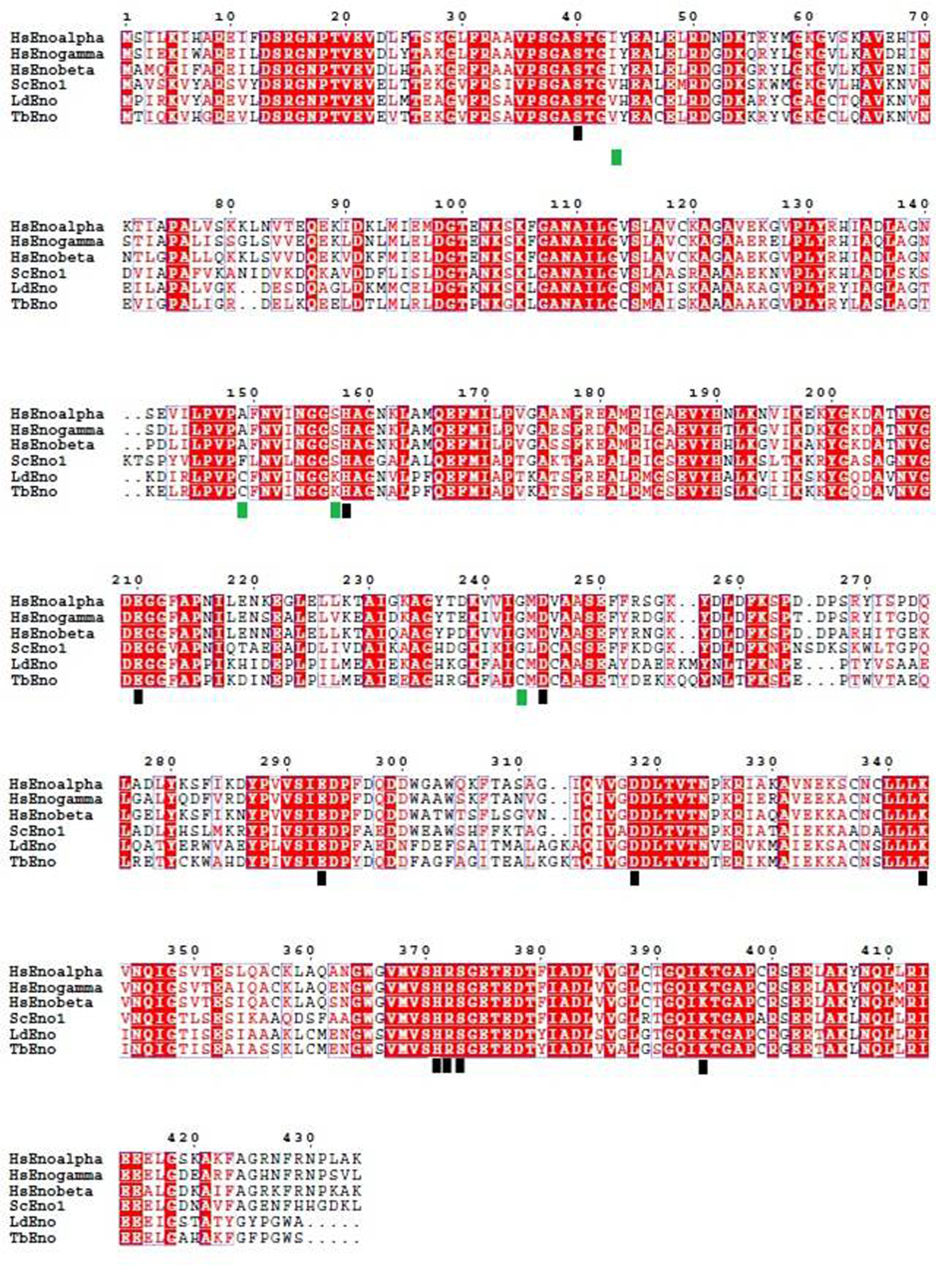

Supplement: Figure S3 — Multiple sequence alignment of enolase sequences of Leishmania donavani (LdEno; ACE74540.1), Trypansoma brucei (TbEno; Q9NDH8), Saccharomyces cerevisiae (ScEno; P00924), Human (HsEnoalpha; P06733, HsEnobeta; P13929 and HsEnogamma; P09104). Fully conserved active site residues are shown by black rectangular dots, while residues near to active site which differs from Human enolase, are shown in green rectangular dots. Alignment image was produced by ESPript (www.espript.ibcp.fr/ESPript/ESPript/) after submitting aligned sequences generated by ClustalW (www.ebi.ac.uk/Tools/msa/clustalw2/). (TIF) [file pone.0086073.s003.tif]

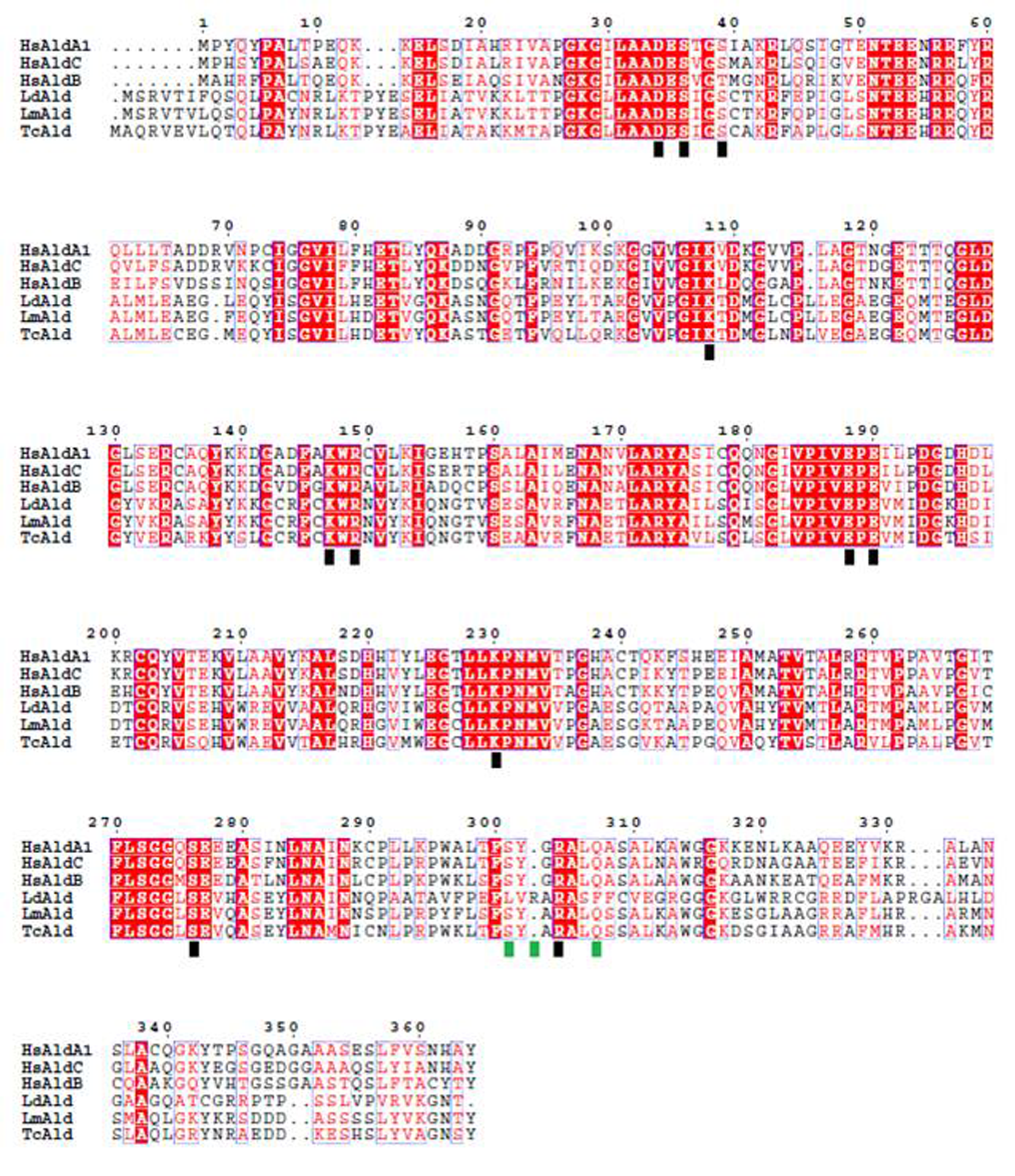

Supplement: Figure S4 — Multiple sequence alignment of aldolase sequences of Leishmania donovani (LdEno; ACT67434.1), Leishmania mexicana (LmAld; CAB55315.1 ) Trypansoma cruzi (TbEno; XP_809370.1) and Homo sapiens (HsAldA1; NP_001121089.1, HsAldB; NP_000026.2 and HsAldC; NP_005156.1). Fully conserved active site residues are shown by black rectangular dots, while residues near to active site which differs from Human enolase, are shown in green rectangular dots. Alignment image was produced by ESPript (www.espript.ibcp.fr/ESPript/ESPript/) after submitting aligned sequences generated by ClustalW (www.ebi.ac.uk/Tools/msa/clustalw2/). (TIF) [file pone.0086073.s004.tif]

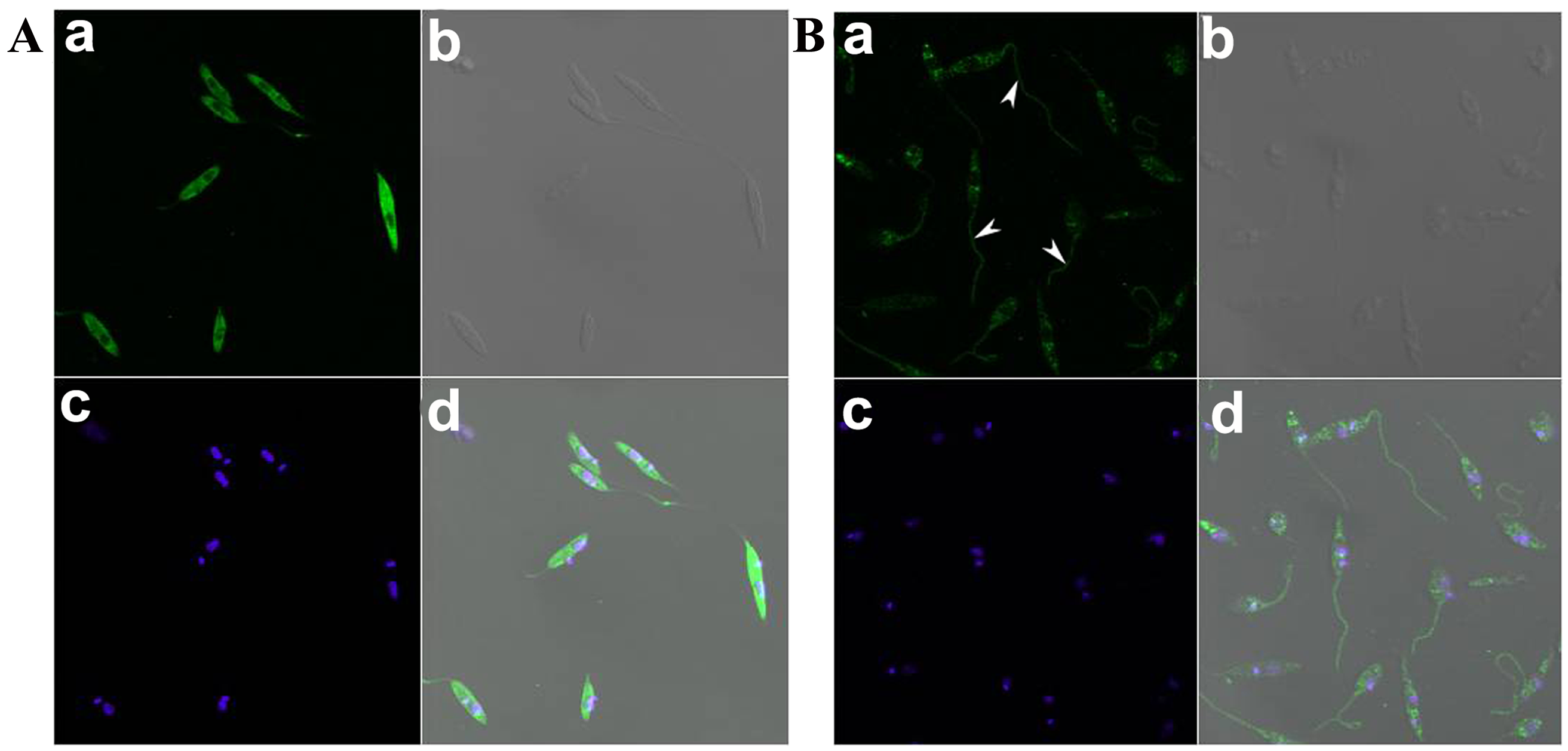

Supplement: Figure S5 — Immunofluorescence analysis of the intracellular distribution of enolase (LdEno) and aldolase (LdAld) in L. donovani promastigotes; (A) The images showing a diffusely pattern of LdEno throughout the cell body with a marked exclusion of the nucleus; (B) The images showing localization of LdAld in the discrete granular compartments, presumably the glycosomes, apart from its localization through the flagellar length. Arrowheads mark the flagellar localization of LdAld. a, immuno-fluorescence images; b, differential interference contrast image; c, nuclei and kinetoplasts labelled with DAPI; d, merged images. (TIF) [file pone.0086073.s005.tif]
